# Octopamine regulates neural circuits in the mushroom body and central complex, influencing sleep and arousal

**DOI:** 10.1016/j.isci.2026.115564

**Published:** 2026-04-02

**Authors:** Martin Reyes, Yi Shen Lee, Maria Muhammad Ali, Preeti Sundaramurthi, Namrata Dhungana, Amanda Nguyen, Thomas Zimmerman, Sara Capponi, Divya Sitaraman

**Affiliations:** 1 Department of Psychology, College of Science, California State University- East Bay, Hayward, CA, USA; 2 IBM Almaden Research Center, San Jose, CA 95120, USA; 3 Center for Cellular Construction, San Francisco, CA 94158, USA

**Keywords:** neuroscience, sensory neuroscience

## Abstract

Sleep is a widespread yet incompletely understood phenomenon, and animals exhibit diverse arousal states beyond the simple binary of sleep and wake. Essential behaviors such as feeding, courtship, and escape often compete with sleep, and biogenic amines—including dopamine, norepinephrine, and serotonin—help regulate these behavioral states across species. Here, we leverage the small number and well-defined connectivity of neuromodulatory neurons in *Drosophila* to investigate how specific octopamine (OA) neurons regulate sleep and arousal. We focus on a pair of OA neurons, VPM3, which project broadly to the mushroom body (MB) and central complex (CX). We find that VPM3 neurons are sexually monomorphic, required for sleep suppression and male courtship, and modulated by sleep history. In addition, the male-specific fruitless isoform in these neurons is necessary for sleep regulation. Combining connectomics with targeted genetic manipulations, we identify key inputs from the CX, MB-mediated downstream pathways, and OA receptor signaling that together reveal how OA circuits coordinate sleep and arousal states.

## Highlights

• A single octopaminergic neuron pair (VPM3) suppresses sleep and promotes arousal
• VPM3 neurons integrate central complex inputs with mushroom body outputs
• Male-specific fruitless expression in VPM3 is required for sleep suppression
• Octopamine receptors in mushroom body circuits mediate VPM3-driven wakefulness

## Graphical abstract

**Figure:**
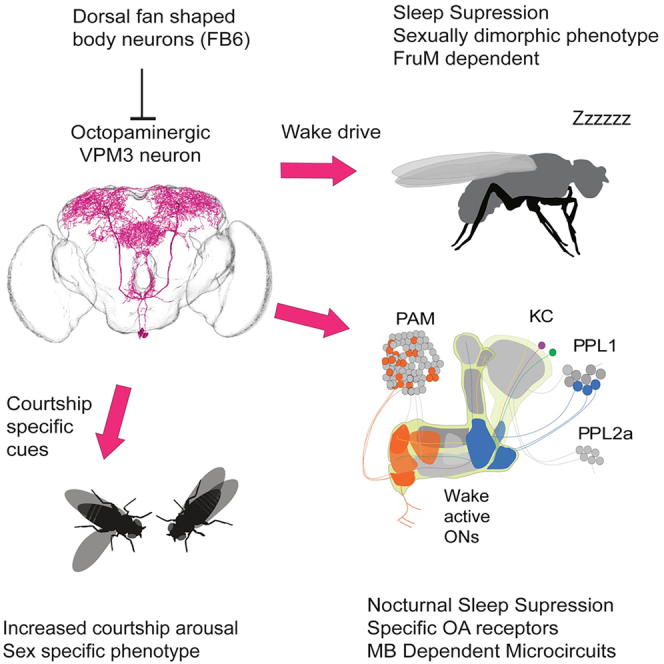

## Introduction

Sleep is a neurobehavioral state that is highly conserved across species and required to maintain physiological and behavioral processes. As a quiescent state sleep prevents animals from engaging in goal-directed behaviors like foraging, mating, and escaping predators that are critical for the survival of the organisms and the species. Hence, conflict between increasing wake hours and activity and need for sleep is continually encoded within the nervous system which in turn allows for these states to persist or break.^1^^,^^2^^,^^3^^,^^4^^,^^5^^,^^6^^,^^7^

The mechanisms that generate sleep, wakefulness, and arousal remain incompletely understood. In flies and mammals, sleep-regulating neurons are distributed across the nervous system, and circuit-level approaches are revealing how these populations integrate state and sensory information to control activity versus quiescence.^8^^,^^9^^,^^10^^,^^11^

Biogenic amines—including dopamine, serotonin, norepinephrine/octopamine, and histamine—modulate diverse functions, from circadian rhythms to motivated behaviors and sleep. In *Drosophila*, relatively few aminergic neurons produce these modulators, but their widespread projections enable state-dependent control across the brain.^1^^,^^3^^,^^4^^,^^7^^,^^12^^,^^13^^,^^14^^,^^15^

In invertebrates, serotonin (5-HT), dopamine (DA), octopamine (OA), and tyramine (TA) are central to sleep regulation. DA and OA generally promote arousal and suppress sleep through multiple circuits. For example distinct DA neurons project to sleep-promoting fan-shaped body (FB) neurons^16^^,^^17^^,^^18^ and wake-promoting mushroom body (MB) neurons.^19^^,^^20^^,^^21^^,^^22^ OA also increases arousal, but comparatively few OA microcircuits are known: ASM OA neurons signal to insulin-like peptide neurons in the pars intercerebralis, whereas male-specific OA neurons (MS1) regulate sleep-arousal conflict. Silencing MS1 neurons reduces female-induced sleep loss and impairs mating behavior.^23^^,^^24^^,^^25^

Here, we focus on a pair of OA neurons, VPM3, with projections in the MB and central complex (CX). VPM3 activity is required for sleep suppression and male courtship, with stronger effects in males. The male-specific fruitless isoform in VPM3 is critical for sleep regulation, and VPM3 activity is shaped by sleep history. Using connectome data and targeting tools, we define the VPM3 microcircuit, identifying CX inputs and MB-dependent outputs that link sleep control across these regions.

## Results

### OA VPM3 neurons promote wakefulness

The MB contains ∼2,000 Kenyon cells (KCs) classified by projections to the α/β, α′/β′, and γ lobes, which receive dopamine, APL, and DPM inputs. MB output neurons (MBONs) tile lobe compartments, and compartment-specific MB circuits regulate sleep.^26^^,^^27^^,^^28^ Dopaminergic PAM and PPL neurons project to sleep- and wake-promoting MB compartments and are essential for state switching and wake maintenance.^19^^,^^20^^,^^21^^,^^22^^,^^26^^,^^29^^,^^30^^,^^31^^,^^32^ Octopamine (OA) neurons also innervate MB, specifically OA-VPM3 and OA-VPM4 broadly project to MB and synapse onto KCs, dopamine neurons (DANs), and MBONs, while OA-VUMa2 targets KCs in the calyx.^33^^,^^34^ Pan-OA activation (Tdc2-GAL4) suppresses sleep, suggesting OA→MB pathways potentially modulate sleep.^23^^,^^35^

**Figure 1:**
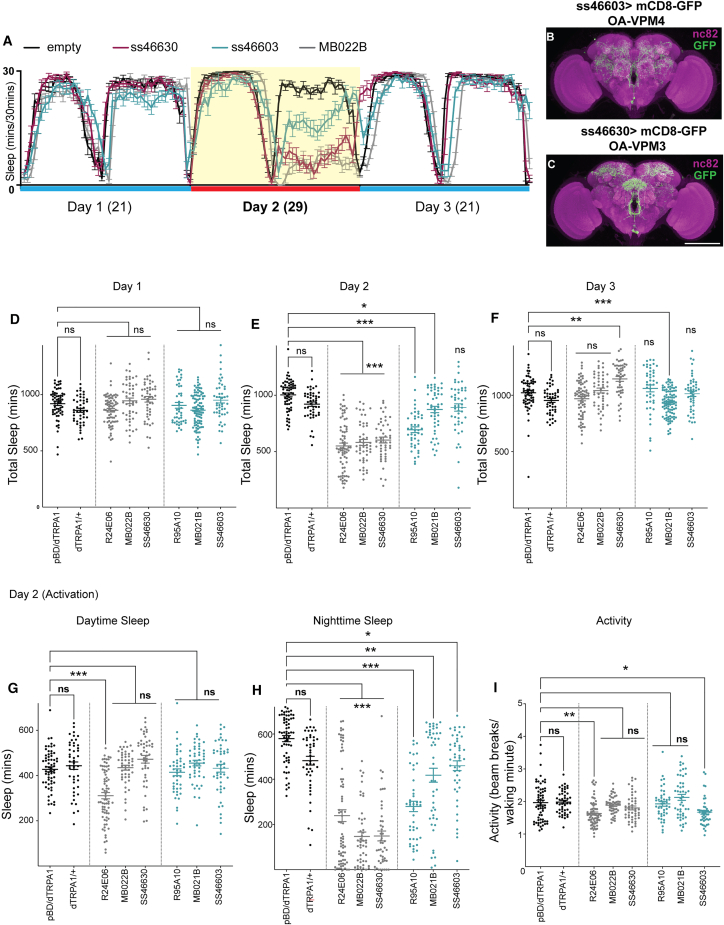
Activation of OA-VPM neurons promotes wakefulness and arousal (A) Sleep profiles of SS46630 (VPM3, red)>UAS-dTRPA1, SS46603 (VPM4, blue)>UAS-dTRPA1, MB022B (VPM3 and 4, gray)>UAS-dTRPA1, and pBD (empty split-GAL4, black)>UAS-dTRPA1. Sleep amount plotted in 30-min bins and profile represents 3 days, 12 h light and 12 h dark condition (day 1: 21, day 2: 29°C (activation) and day 3: 21). (B and C) Whole-mount brain immunostaining of SS46603 (VPM4)>UAS-mCD8-GFP and SS46630 (VPM3)>UAS-mCD8-GFP flies with anti-GFP (green) and anti-Bruchpilot (BRP, nc82, magenta) antibody staining. Maximal intensity projection of the central brain is shown. Scale bars, 100 μm. (D–F) Sleep duration on days 1, 2, and 3 of flies expressing dTRPA1 in broad and specific VPM3 drivers: SS46630 (*n* = 47), MB022B (*n* = 48), R24E06 (*n* = 72), VPM4 drivers: SS46603 (*n* = 46), MB021B (*n* = 47), R95A10 (*n* = 44), and genotypic controls (empty-split>UAS-dTrpA1 [*n* = 62] and UAS-dTRPA1/+ [*n* = 47]). (G, H) Sleep duration during daytime and nighttime on day 2 (activation) of the tested genotypes. (I) Activity or number of beam counts/waking minute on day 2 (activation) is shown for the tested genotypes. For (D)–(I), mean ± SEM is shown and comparisons are made using Kruskal-Wallis test followed by Dunn’s multiple comparisons test. (A) and (D)–(I) show male flies. For this and all subsequent figures, statistical significances are indicated as ∗*p* < 0.05; ∗∗*p* < 0.01; ∗∗∗*p* < 0.001; ns, not significant.

We tested multiple GAL4/split-GAL4 drivers targeting VPM3/4 (R24E06 and MB022B), VPM3 (SS46630), or VPM4 (R95A10, MB021B, and SS46603) and activated neurons using UAS-dTRPA1 (29°C). Five of six drivers significantly reduced sleep on day 2 relative to baseline (21°C) (Figures 1A and 1D–1F), with stronger nighttime suppression (Figures 1G and 1H). In these and subsequent behavioral experiments, we used empty split-GAL4 w [1118]; P {p65-AD.Uw} attP40; P{GAL4-DBD.Uw} attP2 as controls which has enhancer less fragments inserted in the same chromosomal location as the split-GAL4s or GAL4s.

VPM4-specific drivers produced modest sleep suppression, but VNC (ventral nerve cord) expression in R95A10 and MB021B confounded interpretation. To circumvent this we generated a VPM4 driver (SS46603) lacking VNC expression that also showed mild sleep-suppression. In contrast, a specific VPM3 split-GAL4 (SS46630) lacking VNC and VPM4 expression robustly reduced nighttime sleep and induced rebound after activation, without increasing waking activity. Some drivers that suppressed sleep had reduced activity per waking minute, indicating sleep loss was not due to hyperactivity. Together, these data identify OA-VPM3 as a key wake-promoting population.

In addition to males, we also tested age matched (3–8 days) female flies expressing dTRPA1 (Figure S1A) and found only a modest decrease in nighttime sleep and no change in sleep rebound post-activation (Figures S1B–S1D). Activity (beam counts/waking minute) were not significantly different between experimental and control groups (Figure S1E).

We systematically compared VPM3 driver (SS46630) expression pattern in males and females expressing GFP and found it to be identical or indistinguishable at the light microscopy level (Figure S2). Further, we also mined the male and female connectome datasets to identify key synaptic partners and found them to be largely identical in terms of top inputs/outputs and synapse numbers (Table S1).^36^^,^^37^^,^^38^^,^^39^ Anatomically, OA-VPM3 neurons represent a single Tdc2+ pair projecting from the anterior ventrolateral esophagus to SMP and SLP, with extensive γ lobe, superior protocerebrum, and FB innervation, including FB6 and other CX neurons.

To further characterize VPM3-specific sleep phenotype, we activated these neurons during nighttime. The sleep plots show day 1 (LD 21), followed by day 2 (LD, 21:29) and day 3 (LD, 21) (Figure S3A). Sleep duration during daytime and nighttime was compared across tested genotypes for all 3 days (Figures S3B–S3D). Both male and female flies lost sleep during activation on day 2 (nighttime, ZT 12–24) and showed sleep rebound on day 3 (daytime, ZT 0–12). The loss of sleep was stronger in males than females as previously observed. To better visualize these changes, we plotted change in sleep to quantify sleep loss (day 2-day 1, nighttime ZT 12–24) when neurons were activated and subsequent period when sleep rebound occurs (day 3-day 2, daytime, ZT 0–12) (Figures S3E and S3F). Although, sleep loss and sleep gain of SS46630/dTRPA1 flies were significantly different from both controls (SS46630/+ and empty/dTRPA1), the phenotype in female flies was much weaker. Activity (beam counts/minute) were not variable across genotypes and conditions as shown in the heatmap (Figure S3G).

In a complementary approach to examine male-female sleep differences under varying environmental conditions and to address potential light masking effects, we modified our 3-day protocol as follows: day 1 (LD, 21°C), day 2 (DD, 29°C), and day 3 (DD, 21°C). In this iteration, VPM3 neurons were activated on day 2 at 29°C under constant darkness (DD). This design allowed us to test whether the absence of daytime sleep suppression observed previously could be explained by light masking effects. The combination of DD and temperature increase on day 2 modifies sleep patterns and duration as shown in sleep plots (Figure S4A) in both controls and experimental groups. Sleep duration on day 1, day 2, and day 3 (ZT for LD and CT for DD 0–12, 12–24) are shown in (Figures S4B–S4G). In day 1 LD baseline condition, there were no significant differences between SS46630/dTRPA1 as compared to control empty/dTRPA1 and SS46630/+ in male and female flies. On day 2 DD 29°C activation day, sleep loss was observed in CT 0–12 in both males and females and stronger suppression at CT 12–24 was observed in males as compared to females (Figures S4D and S4E). On day 3, DD 21°C, male and female flies slept lesser than one of the controls showing no significant sleep rebound (Figures S4F–S4G). Activity (beam counts/minute) were not variable across genotypes (Figures S5A–S5F) but were variable across environmental conditions.

Across conditions, nocturnal sleep suppression was consistent, supporting a central role for OA-VPM3 neurons in wakefulness, with magnitude shaped by both sex and environment. Sexually dimorphic sleep phenotypes are not uncommon as male and female flies have different sleep patterns and octopamine neurons including the VPM subsets have been implicated in sex-specific behaviors like courtship and aggression.^40^^,^^41^^,^^42^^,^^43^ Further, VPM subsets are thought to express male isoform of fruitless (FruM) which might be the basis of sex-specific behavioral modulation by OA neurons.^40^

**Figure 2:**
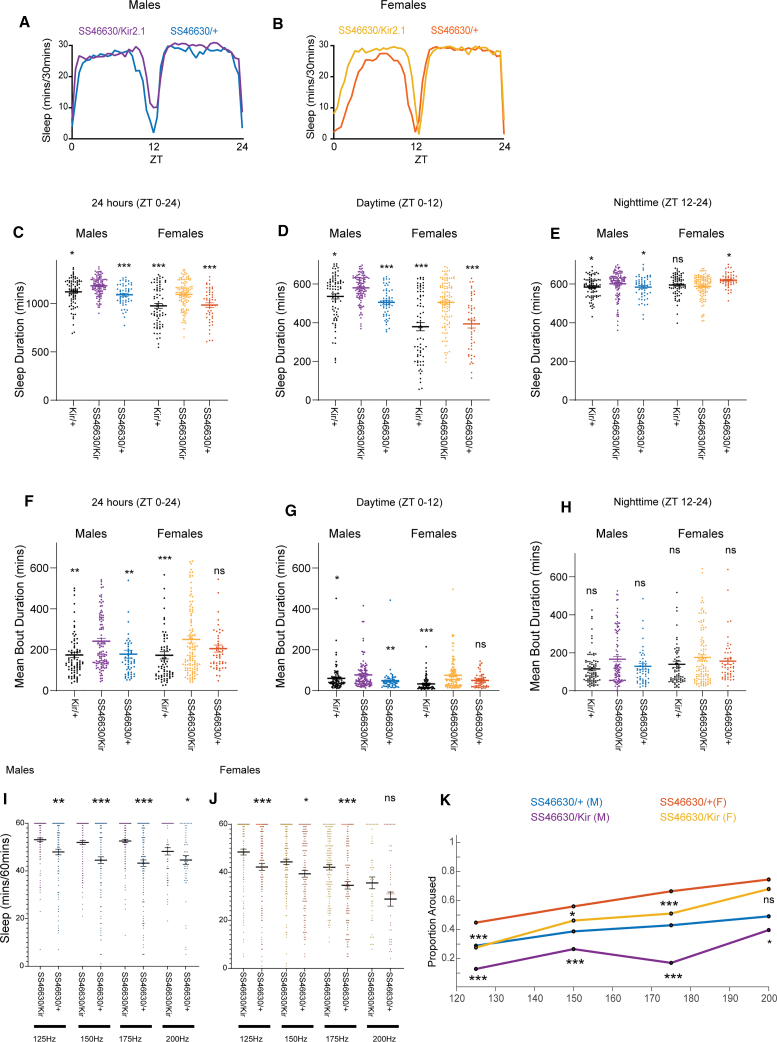
Inhibition of OA-VPM3 neurons increases daytime sleep and bout duration (A and B) Sleep plots (mean) of male and female flies expressing Kir2.1 in VPM3 neurons (SS46630) and controls. (C–E) Total sleep (24 h), daytime (ZT 0–12) and nighttime (ZT 12–24) sleep duration of male and female flies of the genotype SS46630>UAS-Kir2.1, Kir2.1/+, and SS46630/+. (F–H) Mean Bout duration (24 h, daytime ZT 0–12 and nighttime ZT 12–24) duration of male and female flies of the genotype SS46630>UAS-Kir2.1, Kir2.1/+, and SS46630/+. (I and J) Males and female flies SS46630/UAS-Kir2.1 and SS46630/+ were subjected to mechanical stimulation/vibrations at random intervals (totaling 15 min during 60 min recording period). Graphs indicate sleep duration during 60-min interval at different frequencies. (K) Proportion of male and female flies (SS46630/UAS-Kir2.1and SS46630/+) aroused at the four frequencies. Comparisons were made between experimental and control flies for specific frequencies and trendline indicates proportion of flies aroused at different frequencies. For (C)–(G), mean ± SEM is shown, and comparisons are made using Kruskal-Wallis test followed by Dunn’s multiple comparisons test. For (J) and (K), comparisons are made using Mann-Whitney *U* test. Control (SS46630/+ and Kir2.1/+) groups were compared with SS46630/dTRPA1. Number of flies ranged 51–113 for male and female flies.

We next asked whether OA-VPM3 neurons are required for daily sleep. We expressed UAS-Kir2.1 in VPM3 neurons in male and female flies and compared sleep amount and bout duration during daytime (ZT 0–12) and nighttime (ZT 12–24) with genotypic controls (Figures 2A–2H). Inhibiting VPM3 increased sleep duration (Figures 2C–2E) and bout length (Figures 2F–2H) during the day, with smaller nighttime effects.

We also examined probability of transitions between sleep-wake states, P(doze) and P(wake) (Figure S6). P(wake) is the probability of transitioning from inactivity to activity, and P(doze) from activity to inactivity.^44^ In both sexes, VPM3 inhibition decreased P(wake) (Figure S6). P(doze) was unchanged in males but reduced in females. Activity (beam counts/min) did not differ between groups (Figure S6).

To further assess how VPM3 inhibition influences arousal, we subjected male and female SS46630/Kir2.1 (experimental) and SS46630/+ (control) flies to mechanical stimulation as described in.^22^^,^^44^ Stimuli were delivered between ZT 4–8, when both genotypes are relatively quiescent. Flies in DAM monitors received random 2-second sine-wave vibrations (50–200 Hz) totaling 15 min within a 60-min session. DAMs were mounted on a speaker platform with an accelerometer to ensure equal stimulation.

Sleep during stimulation at four frequencies is shown in males and females (Figures 2I and 2J) and proportion aroused in Figure 2K. Male flies where VPM3 neurons are inhibited show reduced arousal/increased sleep at lower frequences (125, 150, and 175 Hz) and have higher number of flies that sleep through vibrations as compared to controls. However, at the highest frequency (200 Hz), the difference in sleep duration and proportion of aroused flies between experimental and control groups is reduced possibly because the stimuli are salient and sufficient to wake up SS46630/Kir2.1 flies. Interestingly, sleep in female flies is more sensitive to the stimuli even at lower frequencies and at the highest frequency both experimental and control flies are aroused.

### VPM3 neurons mediate context-specific arousal

Numerous studies have addressed the role of subsets of octopamine neurons in motivated and goal directed sex-specific behaviors such as courtship and aggression.^25^^,^^40^^,^^42^^,^^45^^,^^46^^,^^47^^,^^48^^,^^49^^,^^50^^,^^51^ Broad and specific manipulation of octopamine neurons regulates sleep but it is unclear if specific OA neurons function as multifunctional hubs or are centers for co-regulation of behaviors. Because sleep is measured in isolated flies, it is difficult to assess how OA-induced sleep loss affects other motivated behaviors.

As an initial test of VPM3 function, we mined a publicly available dataset quantifying 2,204 GAL4 lines using the Fly Bowl assay (^52^https://kristinbranson.github.io/BABAM/). We focused on R24E06, which labels OA VPM3/4 neurons with sparse VNC expression. Activation of R24E06 with UAS-dTRPA1 suppresses sleep (Figure 1) and, in male-female groups, increases male wing extension, attempted copulation, touching, and chasing relative to controls. These courtship-related behaviors were prominent in males, with no significant effects in females.^52^

**Figure 3:**
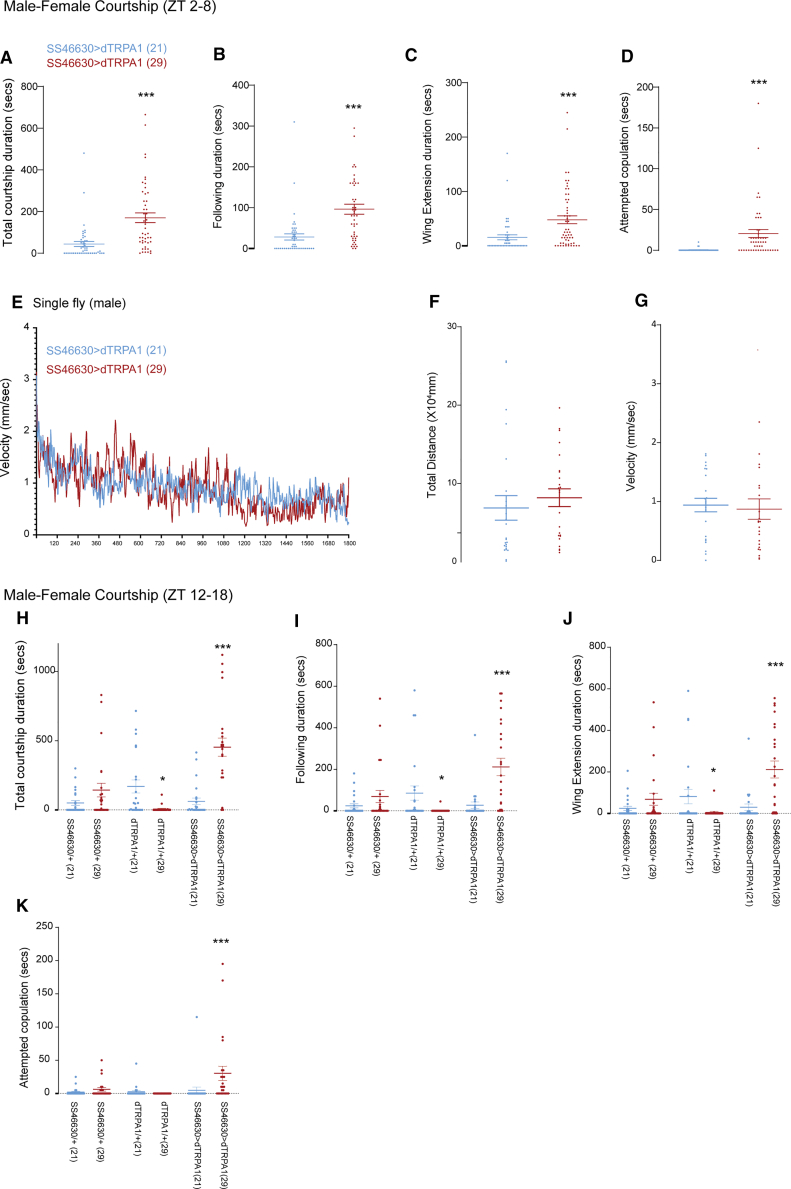
OA-VPM3 activation promotes context-dependent arousal during daytime (ZT2-8) and nighttime (ZT 12–18) (A) Total courtship duration (SS46630>UAS-dTRPA1) during a 30-min recording period (ZT 2–8) at 21°C (blue) and 29°C (red). (B) Total following duration (SS46630>UAS-dTRPA1) during a 30-min recording period (ZT 2–8) at 21°C (blue) and 29°C (red). (C) Total wing extension duration (SS46630>UAS-dTRPA1) during a 30-min recording period (ZT 2–8) at 21°C (blue) and 29°C (red). (D) Total attempted copulation duration (SS46630>UAS-dTRPA1) during a 30-min recording (ZT 2–8) period at 21°C (blue) and 29°C (red). Two conditions were compared using unpaired *t* test (Mann-Whitney *U* test). (E) Velocity vs. time trace of single flies (SS46630>UAS-dTRPA1) during a 30-min recording period at 21°C (blue) and 29°C (red). (F and G) Average distance traveled (SS46630>UAS-dTRPA1) and velocity during a 30-min recording period at 21°C (blue) and 29°C (red). (H–K) Courtship duration and individual metrics (wing extension, following and attempted copulation) of SS46630>UAS-dTRPA1, UAS-dTRPA1/+, and SS46630/+ during a 30-min recording period (between ZT 12–18) at 21°C (blue) and 29°C (red). For each genotype, two conditions were compared using unpaired *t* test (Mann-Whitney *U* test). Number of flies ranged from 24 to 48 for male and female flies.

To test a VPM3-specific role, we activated SS46630>dTRPA1 (VPM3-specific, no VNC expression) between ZT 2–8 and activation significantly increased total courtship duration, following, wing extension, and attempted copulation during 30-min assays (Figures 3A–3D). In the absence of females, SS46630>dTRPA1 males showed no wing extension or courtship behaviors (Figures 3E–3G) and other locomotor measures (distance traveled and velocity) were unchanged, indicating context-dependent arousal.

Because OA-VPM3 activation produces stronger sleep effects at night, we next tested courtship at ZT 12–18, including genotypic (SS46630/+ and dTRPA1/+) and temperature (21°C and 29°C) controls. Arenas were imaged under IR backlight for 30 min and courtship levels were generally higher at night across genotypes. SS46630>dTRPA1 males at 29°C showed increased wing extension, following, and attempted copulation versus 21°C controls. Temperature alone did not induce courtship as dTRPA1/+ males showed greater wing extension and following at 21°C than 29°C, and SS46630/+ controls showed no temperature-dependent differences (Figures 3H–3K). Representative videos are shown in Videos S1 and S2.

Video S1. Sample 30-min video of male-female pairs in dark (ZT 12–18) where male flies are of the genotype SS46630/dTRPA1 at 21°C

Video S2. Sample 30-min video of male-female pairs in dark (ZT 12–18) where male flies are of the genotype SS46630/dTRPA1 at 29°C

Enhanced courtship during both day and night supports a multifunctional role for OA-VPM3 in male arousal. However, increased courtship does not necessarily explain sleep loss, and differences in arenas and conditions limit direct comparisons.

To assess female behavior, we tested SS46630>dTRPA1 females at 21°C and 29°C (ZT 2–8) with tester males (Figure S7). Activation did not alter receptivity measures (pausing, courtship latency, and ovipositor extrusion^53^^,^^54^) consistent with the Fly Bowl results.^52^

Together, these data show that, in addition to suppressing sleep, VPM3 neurons promote courtship arousal in males. Given these strong effects, we focused subsequent analyses on molecular regulators and output pathways in males.

### Sleep suppression by VPM3 is FruM dependent

The expression of the male-specific isoform of fruitless (fruM) is thought to specify the neuronal circuitry required for male courtship behavior.^55^^,^^56^ However, it remains unclear whether fruM expression in specific neurons also contributes to male sleep behavior. We previously showed that FruM knockdown in fruM-positive neurons (fru-GAL4) alters male sleep.^57^ Several octopamine neuron (OA) clusters targeted by Tdc2-GAL4, including VPM neurons, are FruM-positive and regulate sex-specific behaviors.^40^^,^^41^^,^^42^ We, therefore, hypothesized that fruM functions in OA-VPM3 neurons to regulate male sleep.

We co-expressed dTRPA1 and FruM-RNAi in VPM3 neurons and measured sleep at 21°C and 29°C using a 3-day 12:12 L:D protocol, with temperature elevated on day 2. To address UAS dilution (SS46630 driving both transgenes), we included a UAS-GFP control. We compared VPM3 and empty split-GAL4 flies expressing: UAS-dTRPA1(II), UAS-FruM-RNAi (III), UAS-mCD8-GFP(III), UAS-dTRPA1+UAS-FruM-RNAi, and UAS-dTRPA1+UAS-mCD8-GFP.

**Figure 4:**
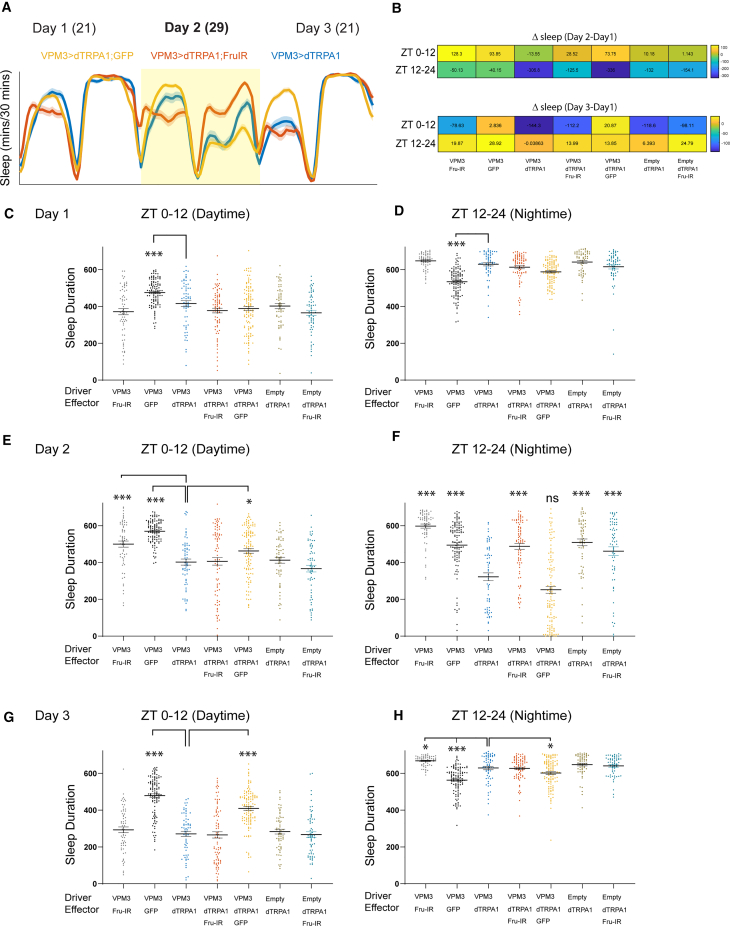
Sleep regulation by OA-VPM3 is FruM dependent (A) Sleep profile of flies (SS46630(VPM3)>dTRPA1, SS46630>dTRPA1; GFP and SS46630>dTRPA1, FruMIR). (B) Heatmap showing change in sleep (means) during daytime (ZT 0–12) and nighttime (ZT 12–24) for day 2-day 1 and day 3-day 1. (C and D) Sleep duration on day 1 (daytime and nighttime) for indicated genotypes with empty-split or SS46630 (VPM3 driver) expressing UAS-dTRPA1 (II), UAS-FruIR (III), UAS-GFP(III), or combinations (UAS-dTRPA1; UAS-GFP and UAS-dTRPA1; UAS-FruIR). (E and F) Sleep duration on day 2 (daytime and nighttime) for indicated genotypes with empty-split or SS46630 (VPM3 driver) expressing UAS-dTRPA1, UAS-FruIR, UAS-GFP, or combinations (UAS-dTRPA1; UAS-GFP and UAS-dTRPA1; UAS-FruIR). (G and H) Sleep duration on day 3 (daytime and nighttime) for indicated genotypes with empty-split or SS46630 (VPM3 driver) expressing UAS-dTRPA1, UAS-FruIR, UAS-GFP, or combinations (UAS-dTRPA1; UAS-GFP and UAS-dTRPA1; UAS-FruIR). In (B)–(H), means ± SEM is shown and groups were compared using one-way ANOVA (Kruskal-Wallis test) followed by pairwise comparisons with SS46630>dTRPA1 (Dunn’s post hoc correction). Mean ± SEM is shown and comparisons are made using Kruskal-Wallis test followed by Dunn’s multiple comparisons test. All genotypes were compared with SS46630>UdTRPA1 and number of flies ranged from 63 to 110.

Sleep plots across 3 days (Figure 4A) and heatmaps of sleep change [(day 2-day 1) and (day 3-day 1)] (Figure 4B) indicate that VPM3 activation induced by dTRPA1 was not affected by co-expression of UAS-GFP using the same driver (SS46630). In contrast, the VPM3-induced sleep suppression was rescued by co-expression of UAS-FruM-RNAi. Sleep duration plots for days 1–3 (12 h blocks) are shown in Figures 4C–4H. Sleep suppression in VPM3>dTRPA1 flies on day 2 was more consistent during nighttime and did not differ significantly from VPM3>dTRPA1; GFP flies. However, in VPM3>dTRPA1; FruM-RNAi flies, this nighttime sleep suppression was reversed and comparable to genotypic controls in which these neurons were not activated.

Day 3 rebound varied across genotypes. The UAS-dTRPA1(II) line here was in a w1118 background, whereas lines in Figures 1 and 3 were outcrossed (w+), which may contribute to differences in sleep loss and rebound. VPM3>dTRPA1 flies primarily lost sleep between ZT 12–18, whereas VPM3>dTRPA1; GFP flies lost sleep throughout ZT 12–24. Although nighttime loss did not differ significantly, differences in ZT 18–24 sleep may explain rebound variation. Activity (beam counts/waking minute; Figure S8) inversely tracked sleep: genotypes with increased sleep showed reduced waking activity and vice versa.

### Sleep need alters VPM3 activity in lateral and medial protocerebrum

Based on GFP expression and EM data (Table S1), VPM3 neurons receive substantial input from CX neurons innervating dorsal FB layers (primarily layer 6; also layers 1, 2, 4, and 5) and project to the MB (KCs, DANs, and MBONs), SMP, and SLP. VPM3 activation increases wakefulness and is followed by rebound sleep in males, though rebound varies with genetic background and conditions (e.g., DD activation).

**Figure 5:**
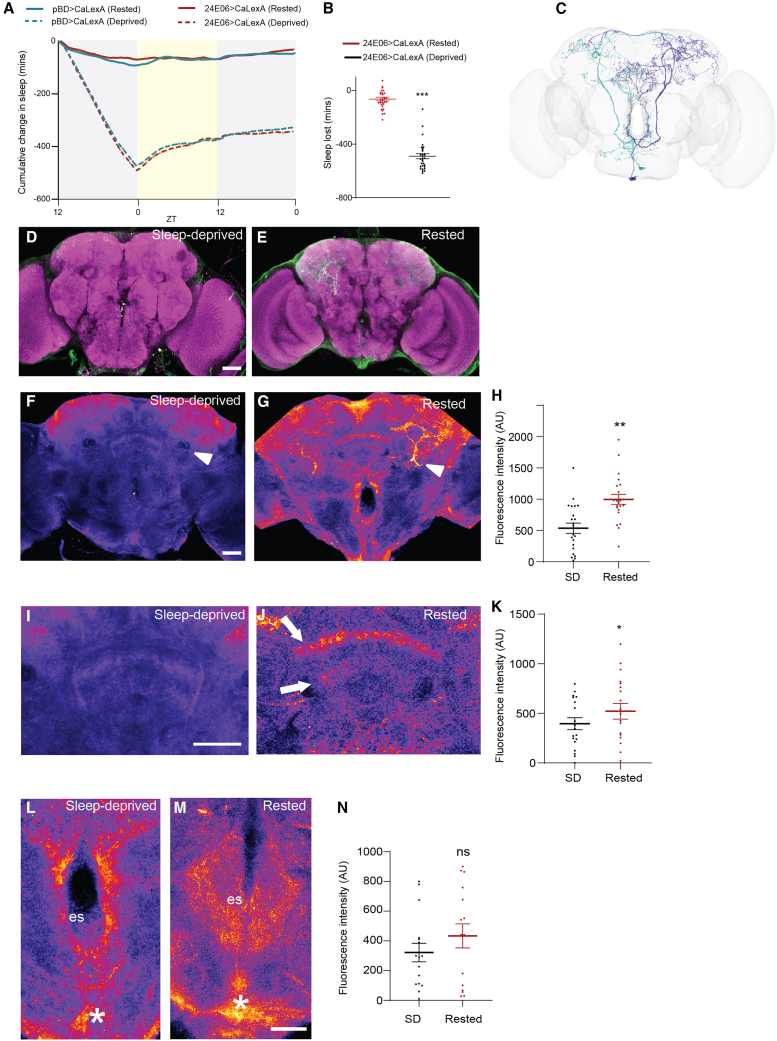
Sleep deprivation alters activity of VPM neurons in central brain regions (A) Cumulative sleep amount in minutes observed 12 h during deprivation and 24-h recovery period (dashed line). Rested flies (solid lines) represent flies that were not deprived and monitored in parallel for the 36-h duration. pBD and 24E06-Gal4 flies expressing CalexA are shown in blue and red, respectively. (B) Quantification of sleep lost (in minutes). Data represent 24E06-CalexA flies that were deprived (sleep deprived) and non-deprived controls (rested). Statistical comparisons are made by Mann-Whitney *U* test. (C) EM-based skeleton reconstruction of OA-VPM3 neurons, projections, and innervated brain regions (adapted from https://codex.flywire.ai/).^36^^,^^37^ (D and E) Whole-mount brain immunostaining of 24E06-GAL4>CalexA flies (sleep-replete controls (B) and sleep-deprived controls (C)) with anti-GFP (green) and anti-Bruchpilot (BRP, nc82, magenta) staining. Maximal intensity projection of the central brain is shown. Scale bars, 100 μm. (F and G) Whole-mount brain immunostaining of 24E06-GAL4>CalexA flies (sleep-replete controls (E) and sleep-deprived controls (F)). GFP staining is pseudo-colored for visualization. Maximal intensity projection of the central brain (10–15 slices, 1 μm) is shown. Arrow indicates region around the peduncle to highlight SMP/SIP projections of OA-VPM3 neurons. (H) Quantification of CaLexA (fluorescence intensity) in the SMP/SIP region in flies (sleep-replete and sleep-deprived). Statistical comparisons are made by Mann-Whitney *U* test, statistical significances are indicated as ∗*p* < 0.05; ∗∗*p* < 0.01; ∗∗∗*p* < 0.001; ns, not significant. (I and J) Whole-mount brain immunostaining of 24E06-GAL4>CalexA flies (sleep-replete controls (E), and sleep-deprived controls (F), CX region are shown. GFP staining is pseudo-colored for visualization. Maximal intensity projection of the central brain (8–10, 1 μm) is shown. Arrows indicate dorsal and ventral projections of FB. (K) Quantification of CaLexA (fluorescence intensity) in the CX region in flies (sleep-replete and sleep-deprived). Statistical comparisons are made by Mann-Whitney *U* test, statistical significances are indicated as ∗*p* < 0.05; ∗∗*p* < 0.01; ∗∗∗*p* < 0.001; ns, not significant. (L and M) Whole-mount brain immunostaining of 24E06-GAL4>CalexA flies (sleep-replete controls (E) and sleep-deprived controls (F)), CX region is shown. GFP staining is pseudo-colored for visualization. Maximal intensity projection of the central brain (8–10, 1 μm) is shown. Asterisks indicate VPM cell bodies and es indicates esophagus. (N) Quantification of CaLexA (fluorescence intensity) in the ventral region (sleep-replete and sleep-deprived). Scale bars, 20 μm. Data included 8–10 male fly brains.

To test whether sleep deprivation alters VPM3 activity, we used CaLexA driven by 24E06-GAL4. CaLexA uses a calcium-dependent reporter: calcium activates calcineurin, which dephosphorylates NFAT, allowing the mLexA-VP16-NFAT transcription factor to enter the nucleus and drive GFP expression via LexAop.^58^ Thus, comparing fluorescence intensity in sleep-replete and sleep-deprived flies allows assessment of how sleep need modulates VPM activity. Sleep deprivation was induced by 12 h of nighttime mechanical shaking, and sleep loss in 24E06-GAL4>CaLexA flies was verified behaviorally; sleep-replete flies served as controls (Figures 5A and 5B). Whole-mount brain GFP fluorescence revealed significantly increased signal in the SMP/SLP region near the peduncle—innervated by MBONs, DANs, and dorsal FB layers—in sleep-deprived flies. Ventral esophageal regions with dense VPM projections were highly variable and did not differ significantly. Because 24E06-GAL4 labels both VPM3 and VPM4, we next used SS46630 to restrict CaLexA to VPM3. GFP signal was weak or undetectable, likely due to lower driver strength. VPM3 and VPM4 overlap in SMP/SLP and MB lobes, but only VPM3 projects to the FB. Although we cannot exclude a role for VPM4, these data indicate that sleep need modulates VPM3 activity in central brain regions.

### VPM3 neurons regulate sleep via interconnected MB input-output pathways

**Figure 6:**
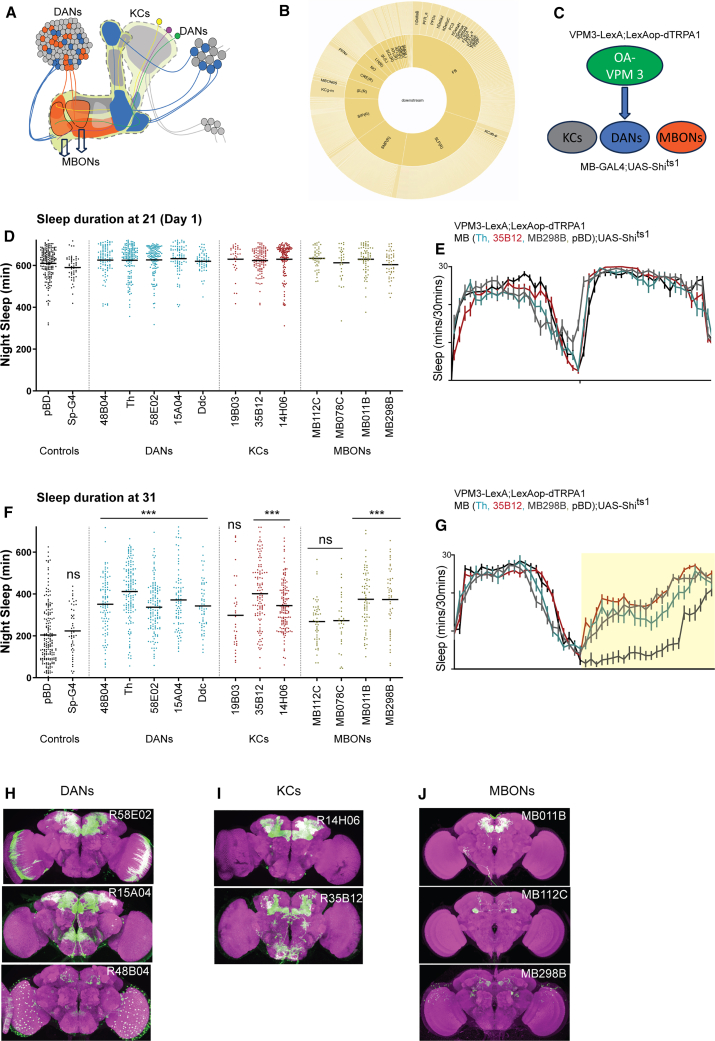
OA-VPM3-dependent arousal is mediated by sleep regulating neurons within MB (A) Schematic of MB KC bodies are represented gray and blue circles depending on the projection lobe. KCs dendrites extend within the calyx and axons project to form α/β, α'/β′, and γ lobes. PAM and PPL1 dopamine neurons (DANs) extensively innervate the lobes. MBONs transmit signals from the MBs to various other brain regions. (B) OA-VPM3 (hemibrain id: 329566174, 5813061260) output represented as a starburst pattern from hemibrain connectome shows extensive downstream signaling via γ lobes, PAM-DANs, and specific MBONs. (C) Experimental strategy to study neuronal pairs by simultaneously activating VPM3 neurons (24E06-LexA; LexAop-dTRPA1) and silencing potential downstream neurons (X-Gal4; UAS-Shi^ts1^). (D) Nighttime sleep duration (day 1, baseline at 21) of flies expressing 24E06-LexA; LexAop-dTRPA1 and X-Gal4; UAS-Shi^ts1^. X-Gal4 includes DANs (48B04 *n* = 99, 15A04 *n* = 83, 58E02 *n* = 134, TH *n* = 147, and Ddc *n* = 58), KCs (14H06 *n* = 138, 19B03 *n* = 32, and 35B12 *n* = 109) and MBONs (MB078C *n* = 32, MB112C *n* = 50, MB298B *n* = 48, and MB011B *n* = 67). Controls include empty-split Gal4 (*n* = 180) and w1118 (*n* = 55). (E) Sleep profile of selected lines showing daytime and nighttime sleep at 21. (F) Nighttime sleep duration (day 2, 31) of flies expressing 24E06-LexA; LexAop-dTRPA1 and X-Gal4; UAS-Shi^ts1^. X-Gal4 includes DANs (48B04 *n* = 99, 15A04 *n* = 83, 58E02 *n* = 134, TH *n* = 147, and Ddc *n* = 58), KCs (14H06 *n* = 138, 19B03 *n* = 32, and 35B12 *n* = 109) and MBONs (MB078C *n* = 32, MB112C *n* = 50, MB298B *n* = 48, and MB011B *n* = 67). Controls include empty-split Gal4 (*n* = 180) and w1118 (*n* = 55). (G) Sleep profile of selected lines showing daytime (21) and nighttime sleep at 31. (H–J) Whole-mount brain immunostaining of DANs (H), KCs (I), and MBONs (J) expressing 10X-UAS-mCD8-GFP flies with anti-GFP (green) and anti-Bruchpilot (BRP, nc82, magenta) antibody staining. Maximal intensity projection of the central brain was made from original z stack files obtained from https://flweb.janelia.org/cgi-bin. In (D) and (F), mean is shown, and comparisons are made using Kruskal-Wallis test followed by Dunn’s multiple comparisons test.

Light microscopy shows VPM3 dendrites in the sub- and peri-esophageal zones with projections to the MB, CX, and other regions^33^^,^^59^ (Figure S2). We used the adult EM datasets (hemibrain, NeuPrint, and male CNS) to define VPM3 connectivity.^38^^,^^39^ Inputs and outputs were ranked by synapse number to identify strong partners (Table S1; Figures 6A and 6B). Top outputs in male and female datasets included two MBONs, several KC class, and several dopaminergic clusters. Strongest inputs arose from dorsal and ventral FB neurons.

Tdc2-GAL4>UAS-syt-eGFP and UAS-Denmark show extensive FB innervation.^60^ To test whether these FB outputs are OA-VPM3, we performed GRASP between dfb neurons (23E10-GAL4) and OA-VPM3 (24E06-GAL4). Reconstituted GFP localized to FB layer 6, indicating direct synapses (Video S3).

Video S3. Whole mount brain z stack imaged at 40X to detect GRASP signals of 23E10-LexA; 24E06-Gal4 in FB6 layers

We focused on MB outputs (Table S1), as MB cell types and sleep roles are well characterized. VPM3 projects to γ KCs, calyces, and MBON5 and MBON11 within γ compartments. Prior activation screens identified γm KCs and MBON5 as wake-promoting.^26^^,^^27^ MBON05 (γ4>γ1γ2) and MBON11 (γ1pedc>α/β) project to other MBON dendrites, supporting multilayer feedforward circuits.^28^^,^^29^ DANs also innervate sleep-regulating MB compartments, suggesting VPM3 modulates multiple MB nodes (Figures 6B and 6C).

To test whether VPM3-mediated wake requires the MB, we performed epistasis (Figure 6C): VPM3 neurons were activated (VPM3-LexA > dTRPA1) while downstream KCs, DANs, or MBONs (GAL4/split-GAL4) were inhibited with Shibire^ts1^. We measured sleep at 21°C (24 h, 12 hL:12hD) on day 1 followed by temperature shift during nighttime to 31°C (12 h light, 21°C and 12 h dark, 31°C) on day 2. We compared nighttime sleep on day 1 (Figures 6D and 6E) and day 2 (Figures 6F and 6G). Flies expressing dTRPA1 in VPM3 neurons were crossed to empty split-GAL4 (sp-GAL4)/empty GAL4 (pBD) as control and showed significant nighttime sleep suppression (Figures 6D–6G).

VPM3 activation with simultaneous inhibition of specific wake-promoting DANs (PAM and PPL; Figure 6H) neurons, KCs (γ and αʹβʹ; Figure 6I) and MBONs 1–5 (Figure 6J) partially repressed VPM3-induced sleep suppression. However, inhibition of MBON 11 (γ1pedc>α/β) and MBON 10 (β′1) which are also downstream of VPM3 based on EM data, did not rescue nighttime sleep suppression induced by OA-VPM activation. Taken together, VPM3 mediates wakefulness directly or indirectly via wake promoting KCs, DANs, and specific MBONs. Expression patterns of DANs, KCs, MBONs, and VPM3-LexA are shown in Figures 6H–6J (female brains) and Figure S9 (male brains).

In addition to selecting specific driver lines for downstream neurons based on EM data, we also used a different approach to find sleep-regulating neurons downstream of OA-VPM3. A previous study aimed at identifying octopamine receptor expressing neurons involved in aggression generated 34 GAL4 driver lines containing molecularly defined *cis*-regulatory modules (CRMs) for four known *Drosophila* octopamine receptor genes.^51^ Interestingly one of the GAL4 lines we used to target PAM neurons (R48B04) in Figure 6 contains OAMB *cis*-regulatory module and was developed as part of the 34 GAL4 collection targeting octopamine receptor neurons.

Of the 33 GAL4 drivers, we selected 25 that had somewhat sparse expression and minimal VNC expression. We first screened these 25 GAL4 lines (Figures S10A and S10B) with dTRPA1 for sleep phenotypes at 21°C and 29°C. Total sleep at 21°C was not significantly different between tested genotypes but several lines had increased and decreased sleep duration at 29°C as compared to genotypic controls (Figure S10). We found three octopamine receptor GAL4 lines that increased sleep when activated and 7 driver lines that suppressed sleep. We selected eight driver lines that were wake promoting and had broad expression in neuropils innervated by OA VPM3 neurons (Figure S10) and performed cellular epistasis experiments described above.

Nighttime sleep suppression induced by VPM3 activation was inhibited by three octopamine receptor GAL4 lines (R21B06, R50A06, and R19G08; Figures S10C–S10J). R21B06 and R50A06 have localized expressions in KCs, while R19G08 has expression in the FB layers. Taken together, the unbiased screening of octopamine receptor GAL4 lines and cellular epistasis experiments provides converging evidence to support a role for KCs and FB neurons in VPM3-mediated sleep. However, these lines, specifically R19G08 and R50A06 had widespread expression beyond KCs and dfb neurons and were not used for subsequent activation-based experiments.

### Role of specific octopamine receptors in sleep regulation

We next examined specific octopamine receptors in sleep regulation. Because activation of CRM-derived lines from Oamb, Octβ1R, and Octβ2R altered sleep, we tested whether these receptors are required for sleep control. We used two approaches: (1) screening null/hypomorphs for individual octopamine receptors and (2) pan-neuronal knockdown using receptor-specific RNAi. In both cases, we used validated nulls/hypomorphs and RNAi lines confirmed by qRT-PCR and behavioral assays.^61^^,^^62^^,^^63^^,^^64^^,^^65^^,^^66^^,^^67^^,^^68^^,^^69^^,^^70^^,^^71^^,^^72^

**Figure 7:**
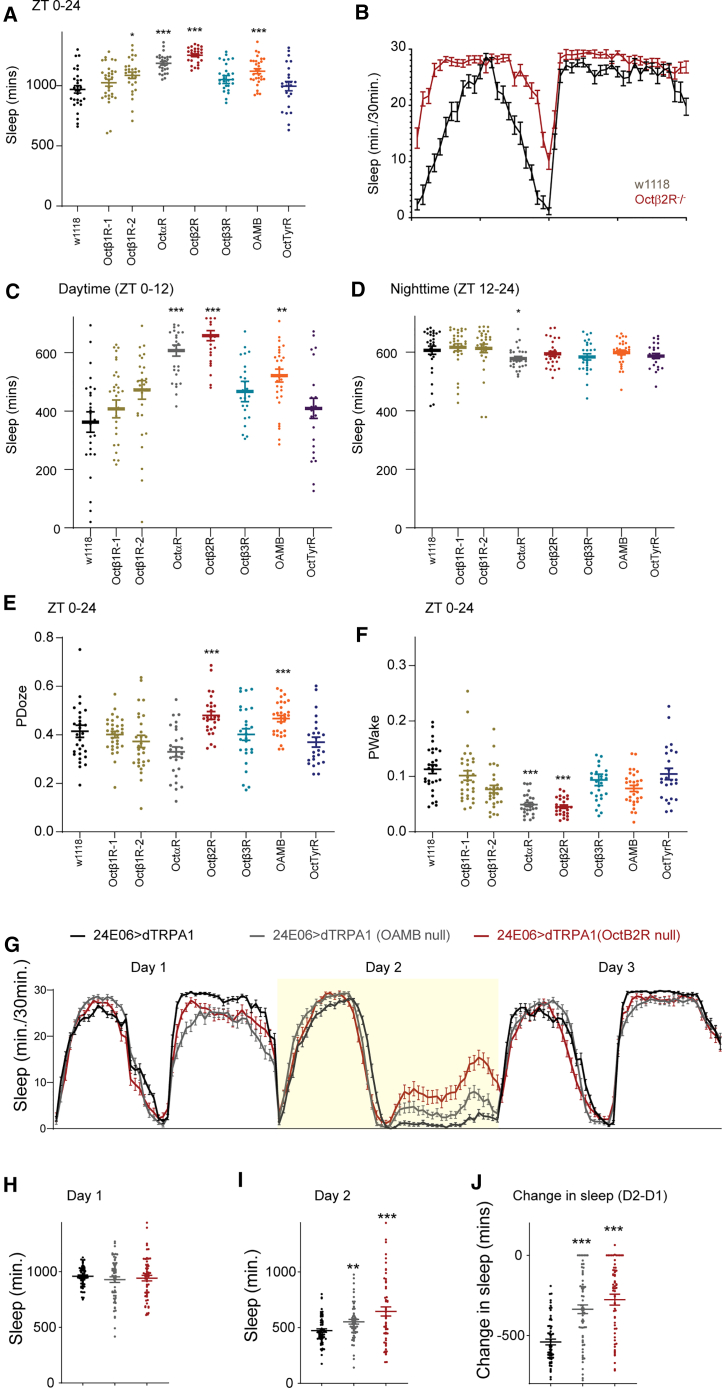
Specific octopamine receptors are involved in sleep regulation and block OA-VPM-induced sleep loss (A) Sleep duration of null/hypomorphs of octopamine receptors (Oct/tyrR [purple] 38558, Oamb [orange] 84555, Octα2R [gray] 18659, Octβ1R-1 [green] 18589, Octβ1R-2 [green] 84554, Octβ2R [red] 18896, and Octβ3R [blue] 43050) are shown. w1118 (black) is used as a genotypic control and data represents mean and SEM and comparisons are made by Kruskal-Wallis test followed by Dunn’s multiple comparisons test. (B) Representative sleep plot (mean and SEM) of Octβ2R^−/−^ (red) and w1118 (black) flies. (C and D) Sleep duration during daytime (ZT 0–12) and nighttime ZT (12–24). (E and F) P(wake) and P(doze) of null/hypomorphs of 5 octopamine receptors (Oct/Tyr [purple], Oamb [orange], Octα2R [gray], Octβ1R [green], Octβ2R [red], and Octβ3R [blue]) are shown. w1118 (black) is used as a genotypic control and data represents mean and SEM and comparisons are made by Kruskal-Wallis test followed by Dunn’s multiple comparisons test. (G) Sleep profile of flies expressing UAS-dTRPA1 in OA-VPM neurons (24E06-GAL4) in Octβ2R and OAMB heterozygous null background (day 1: 21, day 2: 29 (activation), and day 3: 21). (H and I) Sleep duration on day 1 (21) and day 2 (29) of 24E06-GAL4>UAS-dTRPA1 (black), 24E06-GAL4>UAS-dTRPA1 in OAMB heterozygous null (gray), and Octβ2R background (red). (J) Change in sleep induced by activation of OA-VPM neurons (day 2-day 1). Sample size ranged from 57 to 64 flies.

We screened octopamine receptor nulls/hypomorphs outcrossed six generations into w1118. Oamb, Octβ1R, and Octβ2R mutants showed increased sleep versus controls (Figures 7A and 7B), primarily during daytime (Figures 7C and 7D).

We measured P(doze) and P(wake). P(doze) was elevated in one Oamb mutant and in Octβ2R mutants, indicating increased sleep pressure. In these lines, P(wake) was reduced relative to controls and other mutants (Octβ1R and Octβ3R), suggesting increased sleep depth (Figures 7E and 7F).

To test receptor contributions to OA-VPM-mediated arousal, we activated OA-VPM neurons (R24E06>dTRPA1) in Oamb and Octβ2R null backgrounds. Activation-induced sleep loss was partially suppressed in both, more strongly in Octβ2R mutants (Figures 7G, 7H, and S11). Similarly, selective VPM3 activation (SS46630>dTRPA1) in the Octβ2R null background partially rescued sleep loss. All dTRPA1 lines were in w1118; test genotypes were heterozygous for Octβ2R or Oamb.

**Figure 8:**
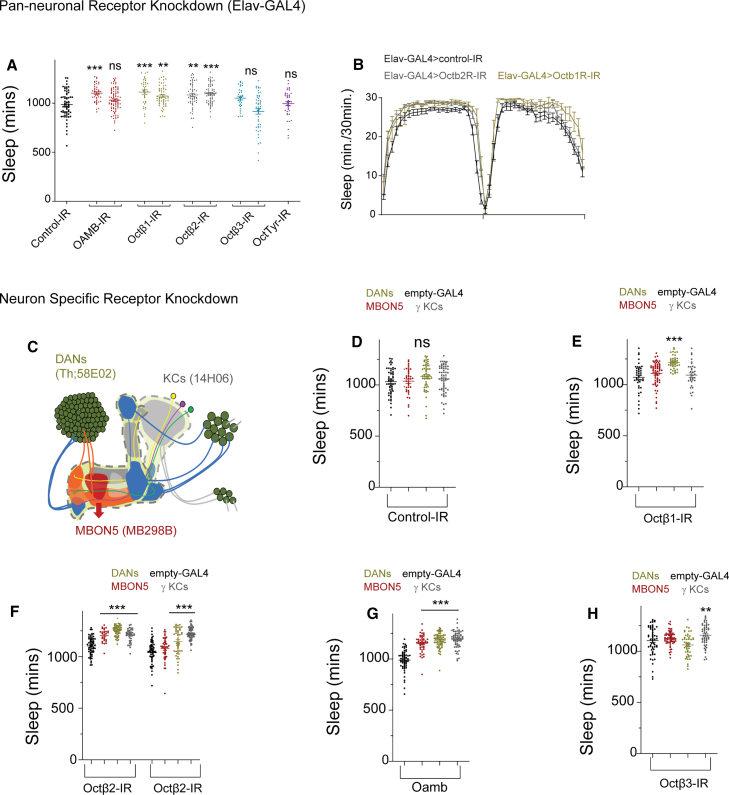
Targeted knockdown of specific octopamine receptor within MB cell types downstream of OA-VPM3 neurons increases sleep duration (A) Sleep duration of flies expressing elav-GAL4 and octopamine receptor RNAi lines. Oct/tyrR (purple, 28332 [*n* = 63]), Oamb (red, 31233 [*n* = 43] and 31171[*n* = 82]), Octβ1R (green, 58179 [*n* = 37] and 50701 [*n* = 58]), Octβ2R (gray, 50580 [*n* = 54] and 34673 [*n* = 63]), and Octβ3R (blue, 62283 [*n* = 31] and 31108 [*n* = 48]) are shown. Elav-GAL4>36303 (black, empty RNAi) is used as a genotypic control, and data are represented as scatterplots and comparisons are made by Kruskal-Wallis test followed by Dunn’s multiple comparisons test. (B) Representative sleep profile (mean and SEM) of Elav-GAL4>36303 (black), Elav-GAL4>UAS-Octβ2R-IR (red), and Elav-GAL4>UAS-Octβ1R-IR (green). (C) The schematic of MB where KC bodies are represented in gray and blue circles depending on the projection lobe. KCs dendrites extend within the calyx and axons project to form α/β, α'/β′, and γ lobes (targeted by 14H06-GAL4). PAM and PPL1 dopamine neurons (DANs) extensively innervate the lobes (green, Th;58E02). MB output neurons (MBON5), a key OA-VPM3 output is shown in red (MB298B). (D–H) Sleep duration of flies expressing split-pBD (empty-GAL4, black), MB298B (MBON5, red), Th;58E02 GAL4 (DANs, green), 14H06-GAL4 (γKCs, gray) and octopamine receptor RNAi lines (RNAi control [36303], Octβ1R [58179], Octβ2R [50580, 34673], Octβ3R [31108], and OAMB [31233]). Empty-GAL4 expressing specific receptor RNAi line is used as a genotypic control, and data are represented as mean and SEM and comparisons are made by Kruskal-Wallis test followed by Dunn’s multiple comparisons test. Sample sizes ranged from 28 to 64 per genotype.

We next performed pan-neuronal RNAi knockdown (elav-GAL4) of validated Oamb, Octβ1R, and Octβ2R lines (Figures 8A and 8B), with efficacy confirmed by qPCR (Figure S11). RNAi lines that increased sleep pan-neuronally were targeted to MB subsets: KCs (14H06-GAL4), DANs (TH-GAL4; 58E02-GAL4), and MBON5 (MB298B). In MBON5, knockdown of Octβ2R, Octβ3R, and Oamb increased sleep. Similar effects were observed in KCs. In DANs, knockdown of all four receptors (Octβ1R, Octβ2R, Octβ3R, and Oamb) increased sleep (Figures 8D–8H). Together, these data indicate that multiple octopamine receptors act in distinct downstream MB neurons to promote wakefulness. Given the interconnected MB network, octopamine likely engages multiple nodes to drive arousal.

### CX inputs regulate activity of OA-VPM3 neurons

Based on our data, we propose that OA-VPM3 neurons promote arousal, enhance male courtship drive, and suppress sleep by activating wake-promoting MB neurons, including γ KCs, MBON5, and DANs. EM hemibrain data further show that FB6A and FB6C are key reciprocal partners and major inputs to OA-VPM3. However, available targeting tools for dfb neurons have broad VNC expression and lack cell-type specificity; for example, 23E10-GAL4 labels at least nine cell types.^73^ Additionally, VNC neurons labeled by this GAL4 line are sleep-promoting, confounding interpretation of dfb neurons in tracking sleep need. These inconsistencies have prompted re-examination of their precise role in sleep homeostasis.^10^^,^^74^^,^^75^

However, in dfb neurons ATP levels rise after waking and predispose them to heightened oxidative stress linking enforced wakefulness to oxidative stress and altered mitochondrial dynamics.^76^ The body of evidence makes FB6 neurons upstream of OA-VPM3 a critical node to understand how sleep/wake/arousal signals are relayed and that dopamine is not the only neuromodulatory connection to these neurons.

**Figure 9:**
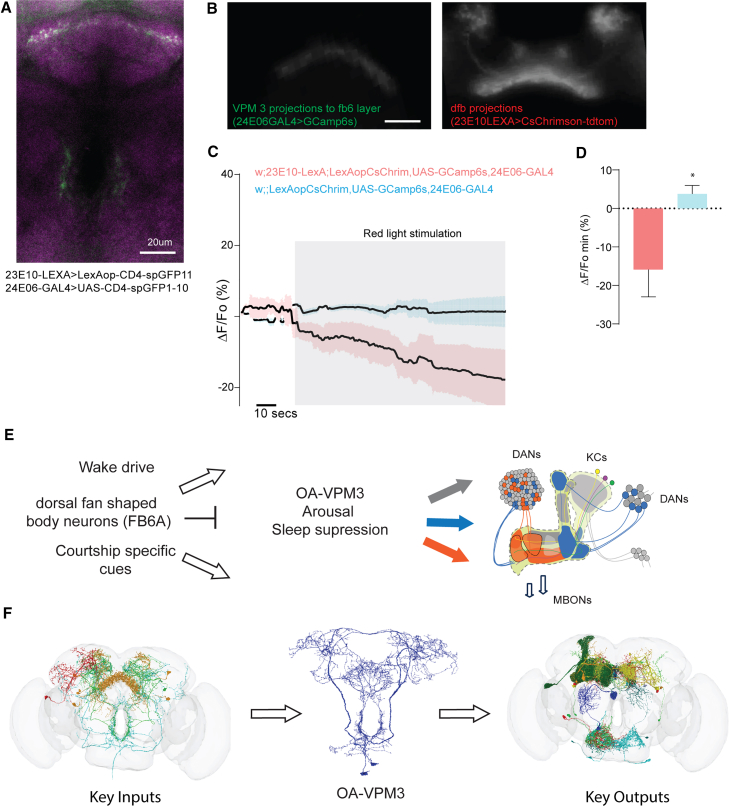
OA VPM3 neurons receive inhibitory input from Fb6 neurons and connect key sleep regulating microcircuits between CX and MB (A) Central section of whole mount of male fly brain showing GRASP, Split-GFP reconstitution across synaptic partners-based connectivity between dorsal fan shaped body neurons targeted by 23E10-LexA and OA-VPM3 neurons targeted by 24E06-GAL4. Maximum intensity projection of the confocal image is shown, and scale bar represents 20 μm. (B) Left and right images showing GCamp6s expression in fb6 layer innervations of OA-VPM3 neurons and dfb (dorsal fan-shaped body projections) of 23E10-LexA expressing CsChrimson-tdTomato. We used this region as a region of interest to measure changes in VPM3 activity as a function of dfb activation in male fly brains. (C) Calcium transient (average traces, mean and SEM) of normalized changes in GCamp6s fluorescence. Red light stimulation was provided 30 s after the start of recording to get a baseline fluorescence. Control group comprised of GCamp6s recording in flies with all transgenes except 23E10-LexA to drive CsChrimson. (D) Bar graphs representing experimental (w,23E10-LexA; UAS-GCamp6s, CsChrimson-tdtomato,24E06-GAL4) and control (w;+; UAS-GCamp6s, CsChrimson-tdtomato,24E06-GAL4). Groups were compared using Mann Whitney *U* test (*p* = 0.014, *n* = 8 brains). (E) Schematic of OA-VPM3’s role in mediating arousal and sleep suppression between CX and MB microcircuits implicated in sleep regulation. (F) Flywire-based filtering (10 or more synapses) of key inputs/outputs of OA-VPM3 neurons.^36^^,^^37^

To test whether dfb neurons (including FB6 classes) excite or inhibit OA-VPM3, we activated dfb neurons (23E10-LexA>CsChrimson) and recorded calcium signals from VPM3 neurons (24E06-GAL4>GCaMP6s). Red light stimulation reduced basal calcium fluorescence in VPM3 (Figures 9B–9D), indicating inhibition. Synaptic contacts in FB layers were confirmed by GRASP (Figure 9A; Video S3).

## Discussion

Although sleep and wake are often treated as binary states, arousal exists along a continuum. Sleep circuits exhibit emergent properties, including multi-timescale integration, nonlinear input-output relationships, and self-sustaining feedback loops,^77^ enabling flexible state transitions.^6^^,^^7^

We focused on OA-VPM3 neurons, which connect with key sleep and arousal centers in the MB and CX. Thermogenetic activation of split-GAL4-targeted OA-VPM3 suppresses sleep, particularly at night, with stronger effects in males. In males, VPM3-induced wakefulness increases subsequent sleep, indicating elevated sleep drive. Consistently, CaLexA-mediated GFP levels show reduced VPM activity after deprivation-induced rebound sleep, supporting a role in sleep homeostasis.

Broad activation of OA neurons (Tdc2-GAL4) promotes wake but suppresses rebound.^35^ In contrast, single-cell targeting reveals that specific OA subsets not only promote arousal but regulate sleep and encode sleep drive. Given their distinct projections, it is unlikely that all Tdc2+ neurons act simultaneously, and broad manipulations may obscure specialized functions within this system. To understand how OA subsets regulate arousal, wakefulness, and sleep drive, we examined VPM neurons in the central brain. OA neurons in this region connect with diverse cell types across multiple neuropils.^78^

The VPM3 subsets are extensively connected with the MB and CX and promote wakefulness via direct and indirect interactions with γ/α′β′-lobes. They are functionally and anatomically linked to PAM DANs and MBON5 and require OAMB and Octβ2R to regulate sleep. OA signaling via OAMB in KCs rescues learning.^62^^,^^79^ OA signaling in α/β KCs is necessary for aversive olfactory learning, and OA signaling in projection neurons is involved in appetitive olfactory learning.^80^ In addition, Octβ2-R in the dopaminergic PPL1 neurons is needed to modulate negative DA signals for sweet taste learning and suppression of Octβ2R in α′/β′ KCs impairs anesthesia-resistant memory.^61^^,^^81^ Taken together, analyzing receptor function in specific sets of KCs, MBONs, and DANs shows OA-VPM neurons form multi-layered connectivity with MB and regulate sleep via multiple microcircuits and require both OAMB and Octβ2R.

This is likely to provide a strategy to efficiently reset the larger MB network and provide redundancy to support states of wakefulness and allow efficient and complete transition between sleep to wake states. More direct evidence for this comes from recent work where quantification of active zones using BrprGFP accumulation shows high level of heterogeneity among KC compartments, especially in γ KCs. In imaging experiments, presynaptic components of OA-VPM3/4 visualized by BrprGFP and nSybCLIP (neuronal synaptobrevin, nSyb; synaptic vesicle marker) show dense localization in γ1 compartments as compared to other compartments and this is, complementary to the Brp compartmental heterogeneity of KCs. In Tβh null alleles, this heterogeneity is lost and only Octβ2R knockdown (using the same RNAi transgene as used in our behavioral experiment) phenocopies these effects. Further, this heterogeneity is lost in sleep-deprived flies.^82^ These results are consistent with our findings that VPM3 output is reduced in sleep-deprived flies, and Octβ2R knockdown in γ lobe and γ1 MBON (MBON 5) is critical for sleep function.

In addition to the MB, OA-VPM3 has extensive innervation in the CX and based on the adult female brain (FAFB) connectome, VPM3 neurons receive significant input from FB6 layers. Like with MBONs, some of these connections are reciprocal. The tangential neurons targeted by GAL4 lines with prominent expression in FB6 layers have been shown to track sleep need and function as an interconnected network with ER5 neurons controlling sleep-wake states.^18^^,^^83^^,^^84^^,^^85^

The sleep promoting dfB neurons form reciprocal connections with wake-promoting dopamine neurons (DANs) potentially implementing a flip-flop circuit motif.^18^^,^^86^ However, the targeting tools used for dfb cell types have extensive expression in VNC neurons which are also sleep-promoting.^10^^,^^87^ These inconsistencies have led to re-examining the precise role of these neurons in sleep homeostasis.^74^^,^^75^ More recent evidence points out a more complex role for these neurons in sleep. Several transcripts associated with ATP synthesis and mitochondrial respiration are up-regulated in dorsal fan-shaped body (dfb) neurons post sleep-deprivation and altering mitochondrial fission and fusion in dfb neurons affects sleep. The connection between aerobic metabolism, mitochondrial dynamics, and sleep in dfb neurons suggests a more complex and nuanced function of sleep.^75^

Female connectome datasets reveal extensive reciprocal connectivity between OA-VPM3 and dfb neurons, particularly FB6A and FB6D. GRASP and calcium imaging confirm these connections in males, and dfb activation inhibits OA-VPM3 activity. EASI-FISH and RNA-seq indicate that FB6 neurons release AstC and subsets co-release glutamate and acetylcholine.^88^ How these transmitters and peptides modulate OA-VPM3 excitability will clarify FB6-VPM3 interactions. We recently reported activation phenotypes of overlapping dfB layer 6/7 tangential cell subsets lacking VNC expression and found that FB6/7 comprise intermingled sleep- and wake-promoting populations.^88^

In addition to fb6, OA-VPM3 neurons are broadly connected with other cell types of CX that include PFGs, hΔK, hΔB, hΔH, etc. although these connections are sparser based on connectome datasets.^60^^,^^73^ VPM3>CaLexA activity is reduced in sleep deprived flies, including some fb projections which is counterintuitive if this circuit was exclusively functioning as a bistable flip-flop circuit between tangential FB6 (sleep-promoting) and OA-VPM3 (wake-promoting) neurons. However, we suspect that some activity changes in fb6 layers are mediated by other connections in these layers such as wake-promoting tangential fb neurons and hΔ neurons.

Sex-specific differences in arousal requirements likely contribute to sexually dimorphic sleep patterns and circuit function. Octopamine circuits are largely sexually monomorphic, and subsets are FruM positive and known to regulate sex-specific arousal behaviors such as aggression, courtship, and egg laying.^25^^,^^40^^,^^41^^,^^42^^,^^45^^,^^46^^,^^47^^,^^48^^,^^49^^,^^50^^,^^62^^,^^65^^,^^66^^,^^69^^,^^70^^,^^71^

Although there is no direct evidence of connectivity between OA-VPM3 neurons and fruitless positive P1 neurons that promote courtship and suppress sleep, the extensive projections of VPM3 neurons in SEZ and LH (lateral horn) region suggests potential involvement in sensory processing of courtship relevant cues. Recent work shows that OA-VPM3 activation promotes sugar reinforced short-term memory and inhibits long-term memory, further artificial and odor (and learning) evoked activity helps the flies prioritize a newer experience over existing ones.^89^ This work along with ours where activation induces a courtship arousal in courtship satiated flies suggests that like nor-epinephrine in mammals, OA gates sensory processing to enact motivated perceptual behaviors.^89^^,^^90^

Acute OA-VPM3 activation may increase sensitivity at early and intermediate sensory nodes to prioritize motivational behaviors, whereas sustained activity promotes wakefulness and arousal. How these neurons regulate sleep and arousal across timescales remains unclear. The small number, discrete organization, and defined connectivity of OA-VPM3 neurons provide a tractable system to study how courtship drive and sleep need are encoded and transmitted. Their extensive reciprocal connectivity and modulation of MB and CX microcircuits suggest a mechanism by which these regions coordinate sleep, arousal, and wakefulness.

### Limitations of the study

Although our genetic tools enabled selective manipulation of OA-VPM3 neurons, most experiments relied on thermogenetic activation or inhibition and therefore may not reflect endogenous firing dynamics. Sleep and arousal phenotypes were also influenced by temperature shifts and light conditions, which may have obscured subtle circuit effects. In addition, females showed weaker and more variable sleep suppression than males, but the mechanisms underlying this sex difference could not be resolved with the approaches used here. Our connectome analysis identified candidate synaptic partners of OA-VPM3 neurons, but these structural predictions require functional validation. Likewise, although receptor mutants and RNAi experiments implicate specific octopamine receptors, pleiotropy and compensatory signaling remain potential confounds. These limitations highlight the need for *in vivo* physiological recordings during natural behavior and for further mapping of upstream and downstream circuits that link OA-VPM3 activity to sleep and context-dependent arousal.

## Resource availability

### Lead contact

Further information and requests for resources and reagents should be directed to and will be fulfilled by the lead contact, Divya Sitaraman (divya.sitaraman@csueastbay.edu).

### Materials availability

This study did not generate new unique reagents.

### Data and code availability

All data reported in this article are available from the lead contact upon request. This study did not generate new code. MATLAB scripts used for behavioral analyses and visualizing data are available from the lead contact upon request. MATLAB code used for delivering sine pulses is available here: https://doi.org/10.5281/zenodo.18471737. Any additional information required to reanalyze the data reported in this article is available from the lead contact upon request.

## Acknowledgments

We thank Dr. Gerry Rubin (Janelia Research Campus, 10.13039/100000011HHMI) for split-GAL4/hemi-driver stocks and shared brain and VNC images. The monoclonal nc82 antibody was obtained from the Developmental Studies Hybridoma Bank (NICHD, 10.13039/100000002NIH; 10.13039/100008893University of Iowa). Several stocks were from the Bloomington Drosophila Stock Center (10.13039/100000002NIH P40OD018537). We thank Sitaraman lab members Steven Buchert, Veronica Ramirez, Bridget Fitzgerald, Roxanne Moghaddam, and Suwei Lin for discussions and suggestions. This work was supported by 10.13039/100000002NIH 2R15GM125073-03 (D.S.) and 3R15GM125073-03S1 (M.R.) from 10.13039/100000057NIGMS, NSF CAREER IOS-2042873 (D.S.), and NSF Center for Cellular Construction STC DBI-1548297 (T.Z. and S.C.).

## Author contributions

All authors contributed to study conception and design. Experimental work and data analysis were performed by D.S., M.R., Y.S.L., M.M.A., P.S., A.N., and N.D.; data visualization and figure preparation were carried out by D.S., M.R., Y.S.L., P.S., and A.N.; the manuscript was drafted by D.S., M.R., Y.S.L., and P.S.; and all authors contributed to review and editing; D.S. supervised the project and acquired funding; T.Z. and S.C. developed and implemented hardware and protocols for arousal threshold experiments, including frequency testing and data analysis. All co-authors have read and approved the final version of the article.

## Declaration of interests

The authors declare no competing interests.

## STAR★Methods

### Key resources table

**Table:** 

REAGENT or RESOURCE	SOURCE	IDENTIFIER
**Antibodies**		

Rabbit polyclonal anti-GFP	Invitrogen	Cat # A-11122; RRID: AB_221569
Mouse anti-nc82	DSHB	RRID:AB_2314866
SlowFade™ Gold Antifade Mountant	Thermo Fisher Scientific	Cat #S36936
Normal Goat Serum	Jackson Immuno Research	Cat #005-000-121
Goat anti-Rabbit IgG (H + L) Secondary Antibody, Alexa Fluor™ 488	Thermo Fisher Scientific	Cat# A11034; RRID: AB_2576217
Goat anti-Mouse IgG (H + L) Secondary Antibody, Alexa Fluor™ 568	Thermo Fisher Scientific	Cat# A11031; RRID: AB_144696

**Experimental models: Organisms/strains**		

w[1118]; P{y[+t7.7] w[+mC] = p65.AD.Uw}attP40; P{y[+t7.7] w[+mC] = GAL4.DBD.Uw}attP2	BDSC	79603
w[1118]; P{y[+t7.7] w[+mC] = GAL4.1Uw}attP2	BDSC	68384
MB021B (w; 24E06-p65ADZp in attP40; 95A10-ZpGdbd in attP2/TM6B)	BDSC	68297
MB022B (w; 24E06-p65ADZp in attP40; TDC2-ZpGdbd in attP2/TM6B)	BDSC	68298
ss46630 (w; 77F03-p65ADZp in attP40; VT058566-ZpGdbd in attP2)	Janelia Research Campus	NA
ss46603(w; VT019391-p65ADZp in attP40/CyOTb; VT058566-ZpGdbd in attP2)	Janelia Research Campus	NA
w[1118]; P{y[+t7.7] w[+mC] = GMR95A10-GAL4}attP2	BDSC	41326
w[1118]; P{y[+t7.7] w[+mC] = GMR24E06-GAL4}attP2	BDSC	48051
w+; P{y[+t7.7] w[+mC] = UAS-TrpA1(B).K}attP16	Janelia Research Campus	NA
w;UAS-fruMIR (III)	Janelia Research Campus	NA
w; P{10XUAS-IVS-mCD8:GFP}su(Hw)attP5	BDSC	32188
w+; P{w[+mC] = UAS-Hsap∖KCNJ2.EGFP}7	Janelia Research Campus	NA
w[∗]; P{w[+mC] = LexAop-CD8-GFP-2A-CD8-GFP}2; P{w[+mC] = UAS-mLexA-VP16-NFAT}H2, P{w[+mC] = lexAop-rCD2-GFP}3/TM6B, Tb[1]	BDSC	66542
w[1118]	BDSC	5905
w[∗]; P{w[+mC] = ple-GAL4.F}3	BDSC	8848
w[1118]; P{y[+t7.7] w[+mC] = GMR48B04-GAL4}attP2	BDSC	50347
w[1118]; P{y[+t7.7] w[+mC] = GMR58E02-GAL4}attP2	BDSC	41347
w[1118]; P{y[+t7.7] w[+mC] = GMR15A04-GAL4}attP2	BDSC	48671
w[1118]; P{w[+mC] = Ddc-GAL4.L}4.3D	BDSC	7010
w[1118]; P{y[+t7.7] w[+mC] = GMR19B03-GAL4}attP2	BDSC	49830
w[1118]; P{y[+t7.7] w[+mC] = GMR35B12-GAL4}attP2	BDSC	49822
w[1118]; P{y[+t7.7] w[+mC] = GMR14H06-GAL4}attP2	BDSC	48667
w[1118]; P{y[+t7.7] w[+mC] = R13F04-GAL4.DBD}attP2 PBac{y[+mDint2] w[+mC] = R93D10-p65.AD}VK00027 (MB112C)	BDSC	68263
w [1118]; P{y[+t7.7] w[+mC] = R53H03-GAL4.DBD}attP2 PBac{y[+mDint2] w[+mC] = R30C01-p65.AD}VK00027 (MB078C)	Janelia Research Campus	NA
w[1118]; P{y[+t7.7] w[+mC] = R14C08-p65.AD}attP40/CyO; P{y[+t7.7] w[+mC] = R15B01-GAL4.DBD}attP2/TM6B, Tb[1] (MB011B)	BDSC	68294
w[1118]; P{y[+t7.7] w[+mC] = R53C03-p65.AD}attP40; P{y[+t7.7] w[+mC] = R24E12-GAL4.DBD}attP2/TM6B, Tb[1] (MB298B)	BDSC	68309
w[1118]; P{y[+t7.7] w[+mC] = GMR19G08-GAL4}attP2	BDSC	48863
w[1118]; P{y[+t7.7] w[+mC] = GMR49H01-GAL4}attP2	BDSC	38711
w[1118]; P{y[+t7.7] w[+mC] = GMR50A06-GAL4}attP2	BDSC	38722
w[1118]; P{y[+t7.7] w[+mC] = GMR21B06-GAL4}attP2	BDSC	49857
w[1118]; P{y[+t7.7] w[+mC] = GMR47A04-GAL4}attP2	BDSC	50286
w[1118]; P{y[+t7.7] w[+mC] = GMR47H03-GAL4}attP2	BDSC	50331
w[1118]; P{y[+t7.7] w[+mC] = GMR47H08-GAL4}attP2	BDSC	50335
w[1118]; P{y[+t7.7] w[+mC] = GMR48A07-GAL4}attP2	BDSC	50340
w[1118]; P{y[+t7.7] w[+mC] = GMR48E03-GAL4}attP2	BDSC	50368
w[1118]; P{y[+t7.7] w[+mC] = GMR50A05-GAL4}attP2	BDSC	38721
w[1118]; P{y[+t7.7] w[+mC] = GMR19H07-GAL4}attP2	BDSC	48867
w[1118]; P{y[+t7.7] w[+mC] = GMR20C11-GAL4}attP2	BDSC	48887
w[1118]; P{y[+t7.7] w[+mC] = GMR21A05-GAL4}attP2	BDSC	48921
w[1118]; P{y[+t7.7] w[+mC] = GMR47C08-GAL4}attP2	BDSC	50298
w[1118]; P{y[+t7.7] w[+mC] = GMR47D08-GAL4}attP2	BDSC	50305
w[1118]; P{y[+t7.7] w[+mC] = GMR49A02-GAL4}attP2	BDSC	50398
w[1118]; P{y[+t7.7] w[+mC] = GMR49C04-GAL4}attP2	BDSC	50415
w[1118]; P{y[+t7.7] w[+mC] = GMR49G05-GAL4}attP2	BDSC	38706
w[1118]; P{y[+t7.7] w[+mC] = GMR68B12-GAL4}attP2	BDSC	39463
w[1118]; P{y[+t7.7] w[+mC] = GMR68C01-GAL4}attP2	BDSC	39464
w[1118]; P{y[+t7.7] w[+mC] = GMR68C02-GAL4}attP2	BDSC	39465
w[1118]; P{y[+t7.7] w[+mC] = GMR68C08-GAL4}attP2	BDSC	39468
y[1] w[∗]; Mi{y[+mDint2] = MIC}Oct-TyrR[MI03485]	BDSC	38558
w[1118]; P{y[+t7.7] w[+mC] = GMR68C10-GAL4}attP2	BDSC	39470
w[1118]; P{y[+t7.7] w[+mC] = GMR24E06-lexA}su(Hw)attP5	Janelia Research Campus	NA
w[1118]; PBac{w[+mC] = WH}Octbeta1R[f02819]/TM6B, Tb[1]	BDSC	18589
w[∗]; TI{RFP[3xP3.cUa] = TI}Octbeta1R[attP]	BDSC	84554
w[1118]; PBac{w[+mC] = WH} Octalpha2R[f03483]	BDSC	18659
w[1118]; PBac{w[+mC] = WH} Octbeta2R[f05679]/TM6B, Tb[1]	BDSC	18896
w[∗]; Mi{y[+mDint2] = MIC}Octbeta3R[MI06217]	BDSC	43050
w[∗]; TI{RFP[3xP3.cUa] = TI}Oamb[attP]/TM6B, Tb[1]	BDSC	84555
w[∗]; Mi{y[+mDint2] = MIC}CG43102[MI04839]	BDSC	38558
P{w[+mW.hs] = GawB}elav[C155]; P{w[+mC] = UAS-Dcr-2.D}2	BDSC	25750
y[1] v[1]; P{y[+t7.7] = CaryP}attP2	BDSC	36303
y[1] v[1]; P{y[+t7.7] v[+t1.8] = TRiP.HMJ22156}attP40	BDSC	58179
y[1] v[1]; P{y[+t7.7] v[+t1.8] = TRiP.GLC01702}attP40	BDSC	50580
y[1] sc[∗] v[1] sev[21]; P{y[+t7.7]v[+t1.8] = TRiP.HMS01151}attP2	BDSC	34673
y[1] v[1]; P{y[+t7.7] v[+t1.8] = TRiP.JF01573}attP2/TM3, Sb[1]	BDSC	31108
y[1] v[1]; P{y[+t7.7] v[+t1.8] = TRiP.JF01732}attP2	BDSC	31233
y[1] v[1]; P{y[+t7.7] v[+t1.8] = TRiP.JF02967}attP2	BDSC	28332
y[1] v[1]; P{y[+t7.7] v[+t1.8] = TRiP.HMJ23640}attP40	BDSC	62283
w[1118]; P{y[+t7.7] w[+mC] = GMR23E10-lexA}attP40	BDSC	52693
w[1118]; P{y[+t7.7] w[+mC] = 13XLexAop2-IVS-CsChrimson.mVenus}attP2	BDSC	55139
w[1118]; PBac{y[+mDint2] w[+mC] = 20XUAS-IVS-GCaMP6s}VK00005	BDSC	42749

**Software and algorithms**		

Graphpad Prism 6	Graphpad Inc.	www.graphpad.com
MATLAB 2021	MathWorks	http://www.mathworks.com/products/matlab/
ImageJ/FIJI	NA	https://imagej.net/software/fiji/downloads
Tracking Software	Janelia Research Campus	https://github.com/cgoina/pysolo-tools

### Experimental model and study participant details

#### Drosophila stocks and husbandry

*Drosophila melanogaster* was used in all the studies. Adult male and female Drosophila melanogaster of ages 4–10 days old were used for all experiments and group housed prior to experiments. All GMR GAL4, Split-GAL4, and LexA lines were obtained from the Bloomington Drosophila Stock Center or Janelia Research Campus. Flies used in experiments are listed in the key resources table. All Stocks and crosses were either reared at 21°C or 25°C and 50% humidity and maintained on a 12h:12h light: dark cycle in standard cornmeal media (https://bdsc.indiana.edu/information/recipes/bloomfood.html, Agar 0.56%, Cornmeal 6.71%, Inactivated Yeast 1.59%, Soy Flour 0.92%, Corn Syrup 7% per liter of food). Adult male and female Drosophila melanogaster of ages 4–10 days old were used for all experiments and group housed prior to experiments. All GMR GAL4, Split-GAL4, and LexA lines were obtained from the Bloomington Drosophila Stock Center or Janelia Research Campus. Flies used in experiments are listed in key resources table.

#### Behavioral experiments

Details for each of the four different assays performed in this paper are listed below. In general, all experiments were conducted in incubators maintained at specified temperature and humidity conditions.

Sleep experiments: Locomotor activity and sleep were recorded with the Drosophila Activity Monitor (DAM2) system from TriKinetics (Waltham, MA, USA). Briefly, 3- to 8-day-old male flies were individually transferred to narrow glass tubes (65 mm × 5 mm) containing 5% sucrose and 1.5% agarose, loaded into the DAM systems, allowed to acclimatize to activity monitors and food for at least 12 h, and monitored for 3–7 days in a 12:12 LD cycle. Data was collected in 1-min bins. Total sleep duration, Pwake, PDoze and activity were extracted from the locomotor data as described in.^91^^,^^92^^,^^93^ Sleep profiles/plots were generated depicting average sleep (minutes per 30 min) for the days of the experiment and maintained in the same tube. For experiments involving neural stimulation temperature and light/dark conditions are described in the figures.

#### Mechanical stimulation setup

A custom-built Fly Shaker apparatus was adapted from^22^ and used to deliver low-frequency vibrational stimuli to experimental chambers. The system consisted of a digital Class-D amplifier (FBHDZVV TPA3116D2, 2.1-channel, Amazon Inc) driving a high-power subwoofer mounted on a rigid platform. Audio stimuli were generated using a laptop and converted into mechanical vibrations through the speaker diaphragm. The amplifier module (FBHDZVV TPA3116D2, Amazon Inc) includes a dedicated subwoofer channel (rated up to 100 W), powered by a regulated 20 V DC supply. Audio input was supplied via a 3.5 mm line-in connection from the laptop, and amplified outputs were routed to the subwoofer (Pyle 15″ PLPW15D subwoofer, 2 kW Amazon Inc). The speaker was mounted using a 3D printed DAM mounting wedge, ensuring rigid anchoring of the monitor to shaker platform. The alignment and mechanical coupling were optimized to provide uniform transmission of vibrational energy across the platform surface. Vibrational output of the Fly Shaker was characterized using an accelerometer (WTVB01-BT50 Smart Vibration Module Arduino, 3-axis Vibration Detector, Amazon Inc) mounted on the speaker cone platform surface to ensure uniform vibration.

Male courtship assay: For daytime courtship experiments (ZT 2–8) four to eight-day-old wild-type CS group housed and mated females were loaded individually into round two-layer chambers (diameter: 1 cm; height: 2.5 mm per layer) as courtship targets, and 4–8-day-old tester males (group housed and mated) were then gently aspirated into the chambers. Recording started after flies were loaded for 30 min using a camcorder (Cannon VIXIA HF R800). Courtship tests were conducted at 29°C and 21°C with genotypic controls. Courtship index, which is the percentage of observation time a male fly performs courtship, was used to measure courtship to female targets, and measured manually and scoring was performed single blind. We focused on three different courtship measures following, wing extension and attempted copulation as described in.^57^

For courtship experiments in the dark, flies were collected between ZT 12–18 and quickly transferred to courtship chambers described above under low light conditions. Assays were back-lit by infrared light-emitting diode (LED) strips (Infrared 850 nm IR LED Strip Light, Waveform lighting). Fly behavior was recorded from above the chambers using a Point Gray BFS-U3-200S6M-C USB 3.1 Blackfly S, Monochrome Camera (Edmund Optics) and Spinview software at 30 fps. Vides in the avi format were manually coded for wing extension, following and attempted copulation. Two samples raw videos are shown in Videos S1 and S2.

Female receptivity assay: Tester females of desired genotypes (4–8 days old, group housed and mated) were aspirated into round two-layer chambers (diameter: 1 cm; height: 2.5 mm per layer) and separated from 4- to 8-day-old wild-type CS males (group housed and mated) until courtship test for 15 min. Flies were recorded for 30 min using a camcorder (Cannon VIXIA HF R800). Courtship tests were conducted at 29°C and 21°C with genotypic controls. Female receptivity, which is the percentage of observation time a female fly is receptive to male directed courtship, was used to measure courtship to male targets, and measured manually and scoring was performed single blind. We focused on three different female measures pausing, ovipositor extrusion and copulation as described in.^57^^,^^94^

Single fly assay: Single male flies of desired genotypes (4–8 days old, group housed and mated) were aspirated into round two-layer chambers (diameter: 1 cm; height: 2.5 mm per layer). Recording started after 15 min to allow fly to acclimatize to the chamber environment and continued for 30 min using a camcorder (Cannon VIXIA HF R800). Activity was measured by tracking the flies in each chamber using the OpenCV module in the Python to analyze the 30-min video and then output XY-coordinate and distance data. The analysis program is available freely on GitHub (GitHub - cgoina/pysolo-tools). Female receptivity and single fly assays were all conducted between ZT 2–8.

#### Immunohistochemistry

Brains or ventral nerve cords of 6–10-day old flies were dissected in cold PBS and fixed in 2% paraformaldehyde for 1 h at room temperature. After fixation, samples were washed thrice with 0.05% PBST (PBS containing 0.05% Triton X-100) for 10 min at room temperature (RT), and then blocked in 5% normal goat serum (NGS) solution for 2 h or overnight at 4°C. After blocking, samples were incubated with the primary antibody solution for two days at 4°C. The dilution ratios for the primary antibodies used in this study were 1:1000 for anti-GFP and 1:30 for nc82. Samples were washed in 0.05% PBST thrice in 10-min blocks and incubated with the secondary antibody solution for two days at 4°C. Finally, the samples were washed three times with 0.05% PBST for 30 min at mounted using SlowFade Gold Antifade Mountant and imaged using Leica SP8 confocal microscope. The following antibodies were used: rabbit polyclonal anti-GFP (1:1000; Invitrogen), mouse anti-nc82 (1:50; Developmental Studies Hybridoma Bank, Univ. Iowa), and cross adsorbed secondary antibodies to IgG (H + L): goat Alexa Fluor 488 anti-rabbit (1:800; Invitrogen) and goat Alexa Fluor 568 (1:400; Invitrogen). Representative images illustrating the expression patterns of each driver were chosen from among 5–7 dissected samples.

#### Confocal imaging

Eight-bit images were acquired using a Leica TCS SP5 laser scanning confocal microscope with a 40×/1.3 numerical aperture (NA) or 20×/0.7 NA objective and a 1-3-μm z-step size. Maximum intensity z-projection images were generated in Fiji, a version of ImageJ software. For CaLexA experiment, we quantified fluorescence signal for a sum of slices within a manually drawn ROI. Number of slices summed, thickness of slices, and ROI dimensions were identical between comparison groups. Background fluorescence intensity adjacent to the ROI was measured and subtracted. For sleep deprivation experiments, flies were mechanically stimulated for 6 s per min (pulses were applied at random) for 12 h using a vortexer mounting plate and multi-tube vortexer (Trikinetics) and brains were dissected within 30 min of being removed from the deprivation set up.

#### Ex-vivo imaging of adult brains

Adult brains were dissected in adult hemolymph like (AHL) saline and placed in a perfusion chamber (PC-H chamber, Siskiyou Inc, OR) in AHL solution (5 mM HEPES pH 7.2, 70 mM NaCl, 20 mM KCl, 1.5 mM CaCl_2_, 20 mM MgCl_2_, 19 mM NahCO_3_, 5 mM trehalose, and 115 mM sucrose). A time series of fluorescence images was acquired using an Olympus BX51W microscope with U Plan Aprochromat 40× water immersion objective. GCamp6s was excited with a 470 nm LED light source (X-Cite turbo multiwavelength system, Excelitas Inc) and images were acquired using ORCA FLASH 4.0 V2 digital CMOS camera. For stimulation of Chrimson, a 633 nm LED line was used, while simultaneously acquiring GCaMP6s fluorescence images with a 470 nm LED to measure changes in Ca 2+. At least 5 independent brain preparations were used for all live imaging experiments and the exact number of cells imaged are indicated in the figures. Changes in fluorescence were calculated as ΔF/F = ((Ft − Fo)/Fo) where Fo is defined as the average background-subtracted baseline fluorescence for the 10 frames preceding red light stimulation. All images were processed and quantified using CellSens (Olympus Inc.) and Fiji (ImageJ).

#### RNA analysis

To test the efficiency of knocking down of Octβ2R RNAi and Oamb RNAi transgenes we used qRT PCR. Flies expressing UAS-RNAi pan-neuronally (Elav-Gal4) were collected and heads were removed using razor blades for RNA isolation. Three independent sets of 30 heads were ground using a pestle (Kimble Disposable pellet pestle) and RNA was isolated using QIAGen RNeasy kit based on manufacturer instructions. 14 μL of total RNA from each sample was reverse transcribed with the iScript cDNA Synthesis Kits (BIO-RAD Inc). Isolated RNA was treated with RNase free DNase to eliminate genomic DNA contamination and reverse transcriptase was added to for a total sample amount of 20 μL. cDNA was then diluted into equal concentration (300 ng/uL) to perform qPCR. Each biological replicate was assayed to identify the expression level of the targeted gene (Octβ2R and Oamb), as well as the internal standard endogenous reference genes (rpl32) using iQ SYBR Green Supermix. 1 μL of each diluted cDNA reaction was used in 10 μL qPCR reaction and a master mix was made and 8 μL transferred into wells of a 96-well plate. 2 μL of working primers were then added into each well and mixed. qPCR thermal cycling and fluorescent data acquisition were performed with a BioRad Cfx96 system for 30s at 95°C followed by 40 cycles between 95°C (15s) and 60°C (30s). After the cycles, a melt curve analysis was performed from 65°C to 95°C with a 0.5°C increment in every 5s. Cq values were measured using CFX Maestro Software. A ΔΔCq method was used to process this data to calculate relative gene expression for the experiment.

Primers used (All primers were diluted to a final concentration of 500 nM in reaction).

rpl32 forward 5ʹ-CCG CTT CAA GGG ACA GTA TC-3ʹ

rpl32 reverse 5ʹ-GAC AAT CTC CTT GCG CTT CT-3ʹ.

DmOctβ2R forward 5ʹ-TCC TGT GGT ACA CAC TCT CCA-3ʹ.

DmOctβ2R reverse 5ʹ-CCA CCA ATT GCA GAA CAG GC-3ʹ.

DmOamb forward 5′-AGTCTAGAGCGGTTATACAGCCGACCTA.

DmOamb reverse 5′-AAGAATTCGGGCGGAGTACAGGACATAA.

### Quantification and statistical analysis

All statistical analyses were performed using GraphPad Prism 8 (GraphPad Software, Inc.). Data were assessed for distributional assumptions of normality. For comparisons between two independent groups with normally distributed data, two-tailed unpaired Student’s *t* tests were used. For comparisons between two independent groups with non-normally distributed data, the non-parametric Mann–Whitney U test was applied. For experiments involving comparisons across more than two groups with normally distributed data, one-way or two-way analyses of variance (ANOVA) were performed as appropriate for the experimental design, followed by Tukey’s multiple comparisons post-hoc tests. When non-normally distributed data involved more than two groups, Kruskal–Wallis tests were used, followed by Dunn’s post-hoc multiple comparisons tests. Sample sizes (number of flies per genotype) and significance based on *p* values are reported in the corresponding figures and figure legends. To aid visualization and transparency of the underlying data distribution, results are presented as dot plots showing individual flies rather than bar graphs.

## Supplemental information

Document S1. Figures S1–S11

Table S1. List of EM predicted inputs and outputs of OA-VPM3 neurons in female (Table 1) and male brains (Table 2), left column shows neuron names and right column indicates number of synapses
