# Supplementary material for: Octopamine regulates neural circuits in the mushroom body and central complex, influencing sleep and arousal
Source: iScience. 2026 Apr 2;29(5):115564. doi: 10.1016/j.isci.2026.115564 (PMC13122709; doi:10.1016/j.isci.2026.115564)
Supplement: Document S1. Figures S1–S11 [file mmc1.pdf]

iScience, Volume *29*

Supplemental information

Octopamine regulates neural circuits in the mushroom body and central complex, influencing sleep and arousal

Martin Reyes, Yi Shen Lee, Maria Muhammad Ali, Preeti Sundaramurthi, Namrata Dhungana, Amanda Nguyen, Thomas Zimmerman, Sara Capponi, and Divya Sitaraman

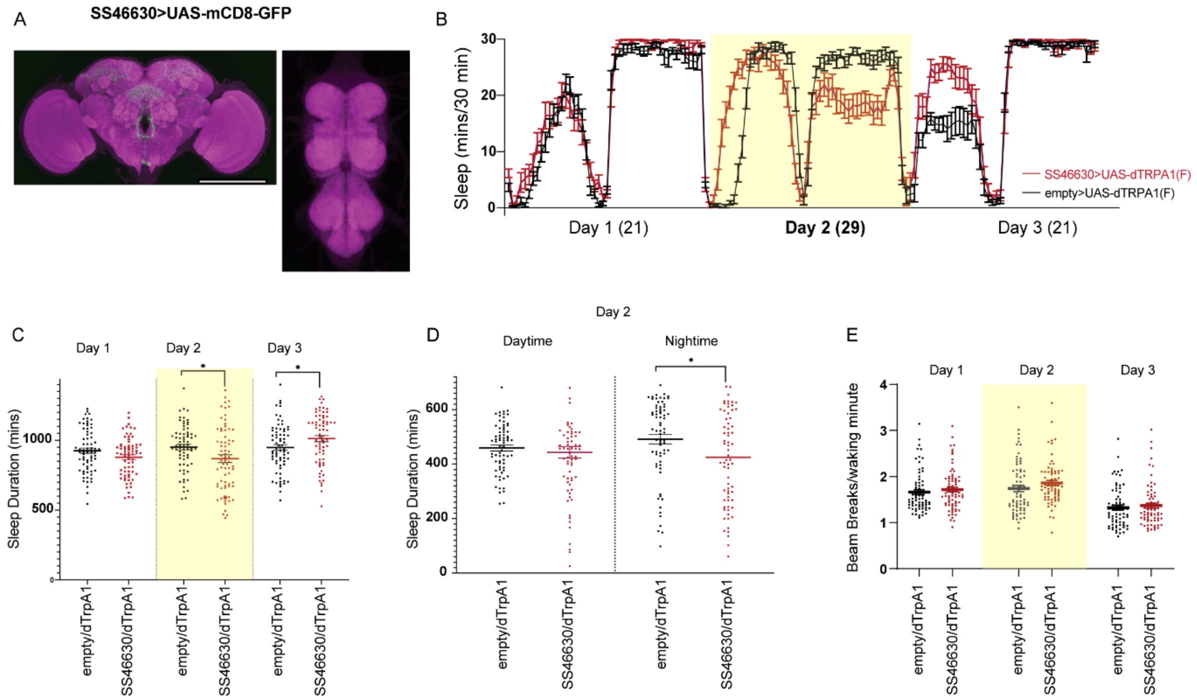

Figure S1: Arousal effects of OA-VPM3 activation are variable between male and female flies.

A. Whole-mount brain and VNC immunostained images of SS46630 (VPM3)>UAS-mCD8-GFP with anti-GFP (green) and anti-Bruchpilot (BRP, nc82, magenta) antibody staining. Maximal intensity projection of the central brain is shown.

B. Sleep plots of ss46630 (VPM3, red)>UAS-dTRPA1 and (empty split-GAL4, black)-split> UAS-dTRPA1. Sleep time of female flies are plotted in 30 min bins and plots represents 3 days, 12h light and 12 h dark condition (day 1: 21, day 2: 29ºC (activation) and day 3: 21).

C. Total sleep duration on days 1, 2 and 3 of female flies ss46630>UAS-dTRPA1 (n=70) and control empty-split>UAS-dTrpA1 (n=70).

D. Sleep duration during daytime and nighttime on day 2 (activation) of the tested genotypes ss46630>UAS-dTRPA1 and empty-split>UAS-dTrpA1. For figures C and D, Mean ± SEM is shown, and comparisons are made using Mann-Whitney U test. Statistical significances are indicated as $*p < 0.05$.

E. Activity or number of beam counts/waking minute on day1, day 2 and day 3 for tested genotypes. Mean ± SEM is shown, and comparisons were made using Mann-Whitney U test.

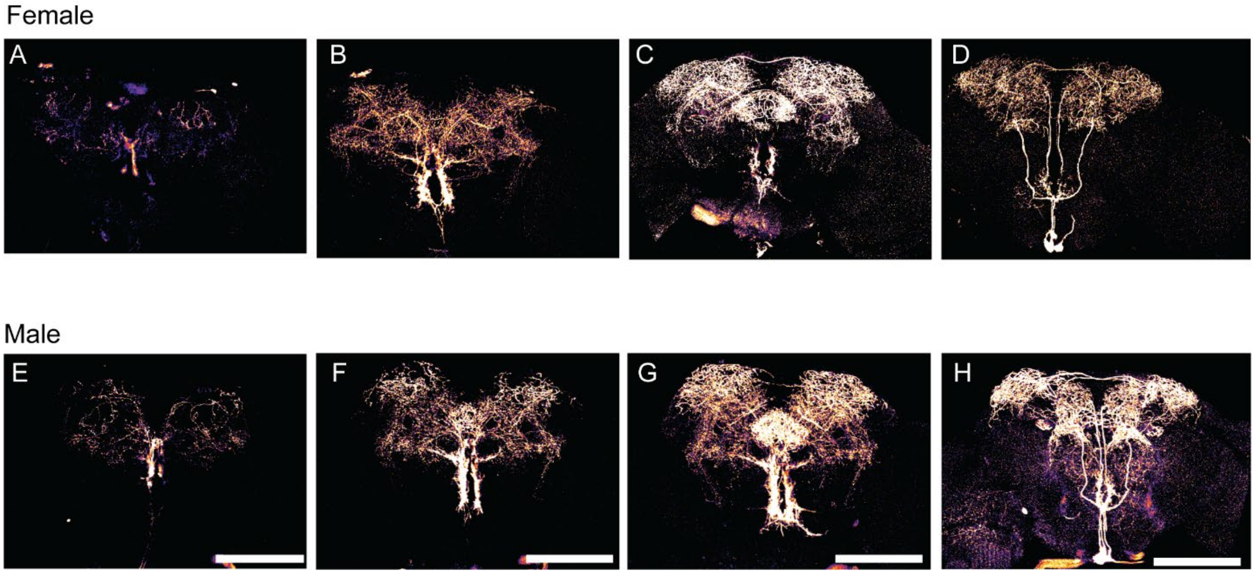

Figure S2: Confocal Imaging of Male and Female brains

A-D: Whole-mount brain immunostaining of ss46630 (VPM3)> UAS-mCD8-GFP female flies with anti-GFP (green) stacks represented from posterior to anterior. Each image is maximal intensity projection of 15-20 (1um slices) pseudo colored to show the extensive innervation patterns within calyx, mushroom body, LH and SLP/SMP.

E-H represents posterior to anterior slices. Scale bar represents 100 um.

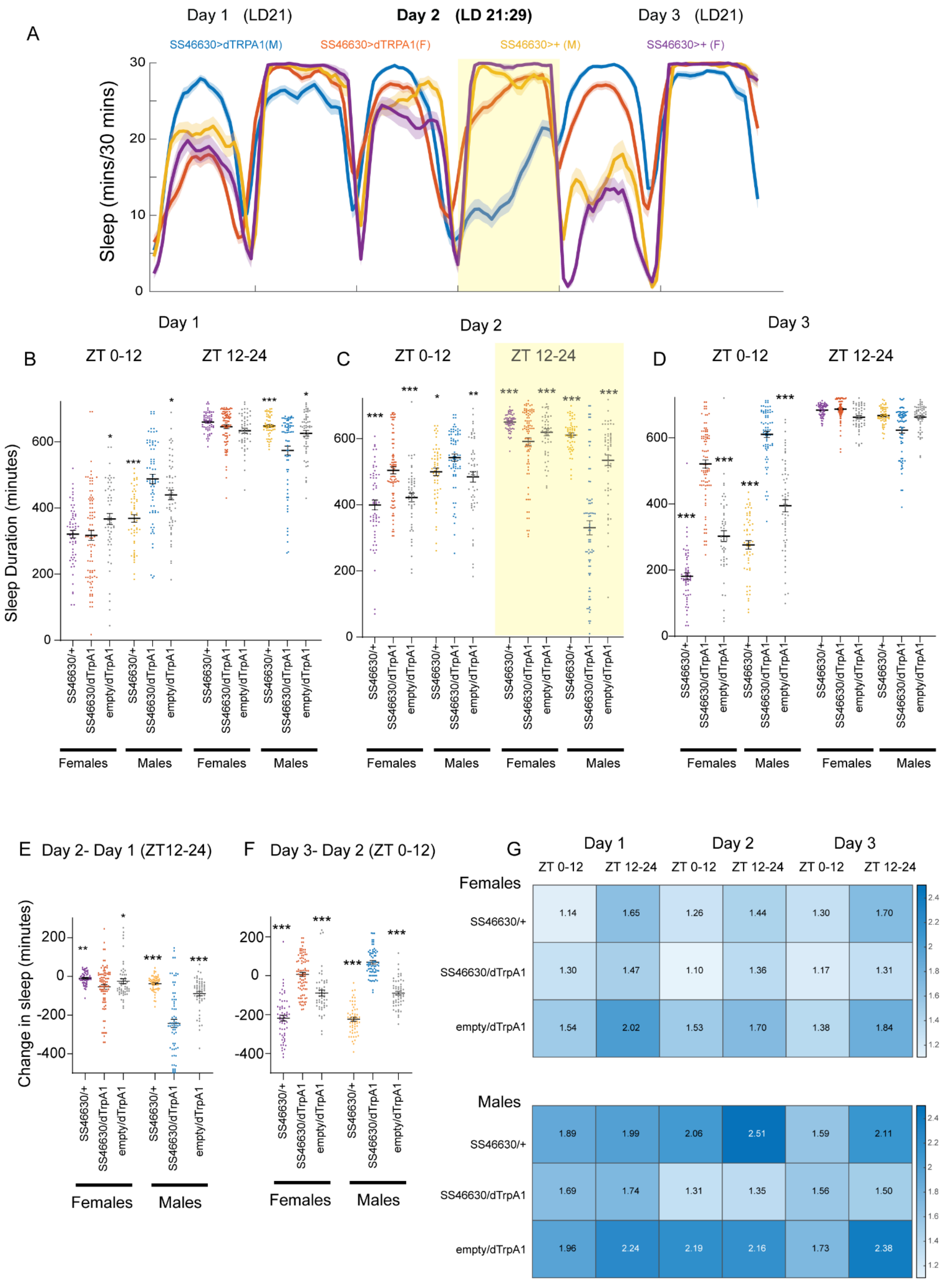
A
Day 1 (LD21)
Day 2 (LD 21:29)
Day 3 (LD21)
SS46630>dTRPA1(M)
SS46630>dTRPA1(F)
SS46630>+ (M)
SS46630>+ (F)
Sleep (mins/30 mins)
Day 1
Day 2
Day 3
B
C
D
ZT 0-12
ZT 12-24
Sleep Duration (minutes)
SS46630/+
SS46630/dTrpA1
empty/dTrpA1
Females
Males
E Day 2- Day 1 (ZT12-24)
F Day 3- Day 2 (ZT 0-12)
Change in sleep (minutes)
G
Females
Males
1.14 1.65 1.26 1.44 1.30 1.70
1.30 1.47 1.10 1.36 1.17 1.31
1.54 2.02 1.53 1.70 1.38 1.84
1.89 1.99 2.06 2.51 1.59 2.11
1.69 1.74 1.31 1.35 1.56 1.50
1.96 2.24 2.19 2.16 1.73 2.38

Figure S3: Nighttime activation of OA-VPM3 neurons suppresses sleep in males and female flies

A. Sleep profile of male and female flies (SS46630>dTRPA1 and SS46630/+) flies.

B. Sleep duration on Day 1 (daytime, ZT 0-12 and nighttime, ZT 12-24) for indicated genotypes with empty-split or SS46630 (VPM3 driver) expressing UAS-dTRPA1 and SS46630/+.

C. Sleep duration on Day 2 (daytime, 21 and nighttime, 29) for indicated genotypes with empty-split or SS46630 (VPM3 driver) expressing UAS-dTRPA1 and SS46630/+.

D. Sleep duration on Day 3 (daytime and nighttime) for indicated genotypes with empty-split or SS46630 (VPM3 driver) expressing UAS-dTRPA1 and SS46630/+.

E and F. Change in sleep Day 2-Day1, Nighttime and Day 3-Day 2, Daytime for indicated genotypes with empty-split or SS46630 (VPM3 driver) expressing UAS-dTRPA1 and SS46630/+.

In figures B-F, means ± SEM is shown and groups were compared using one-way ANOVA (Kruskal-Wallis test) followed by pairwise comparisons of SS46630/+ and empty/dTRPA1 with SS46630>dTRPA1 (Dunns post-hoc correction).

G. Heat map showing activity (beam counts/waking minute) for day 1, day 2 and day 3 (Daytime ZT0-12 and Nighttime ZT 12-24). Top panel represents female flies and bottom panel represents male flies.

Mean ± SEM is shown and comparisons are made using Kruskal-Wallis test followed by Dunns multiple comparisons test. Statistical significances are indicated as ∗p < 0.05; ∗∗p < 0.01; and ∗∗∗p < 0.001. Control (SS46630/+ and empty/dTRPA1) groups were compared with SS46630/dTRPA1. Number of flies ranged 51-87 for male and female flies.

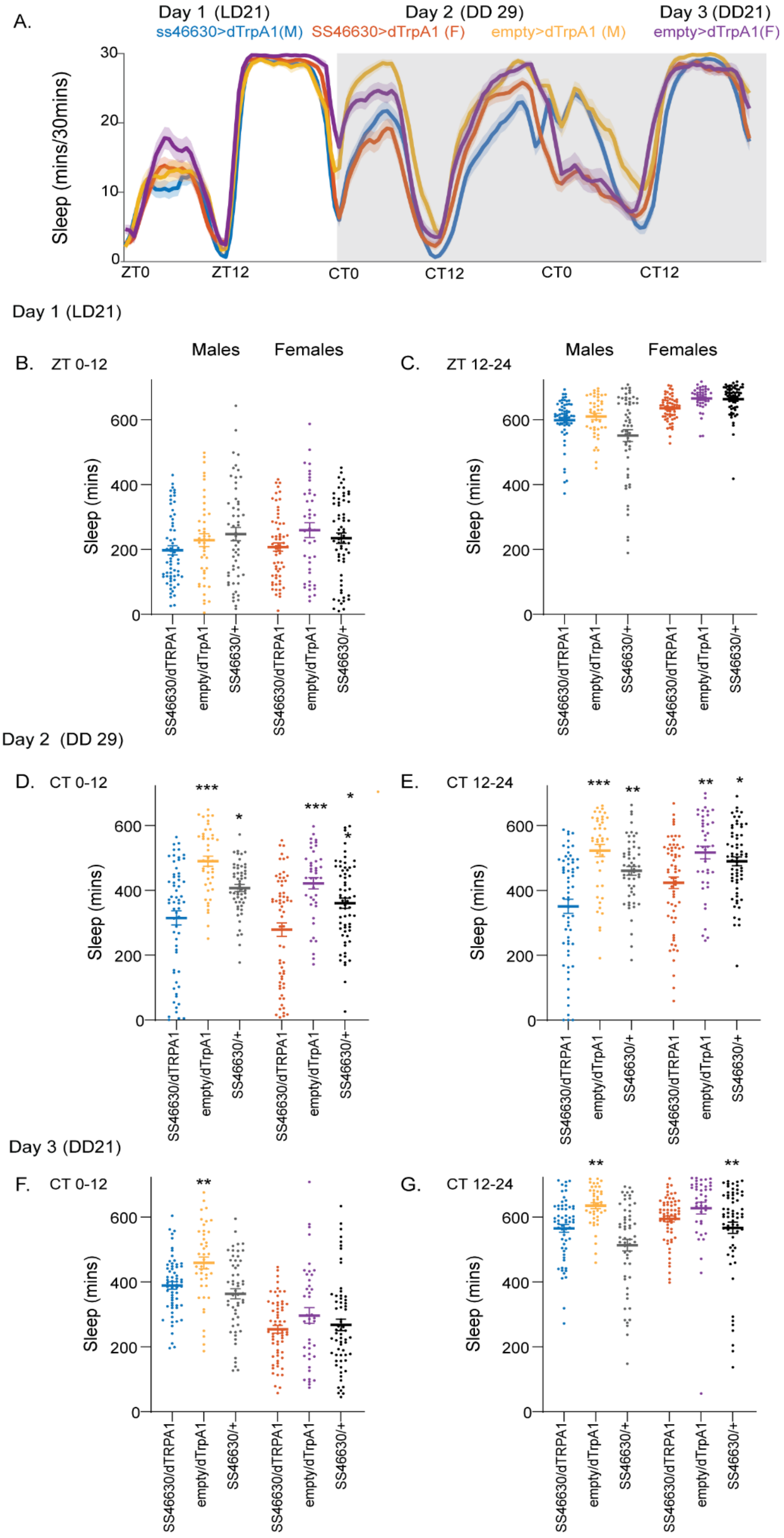
A.
Day 1 (LD21)
Day 2 (DD 29)
Day 3 (DD21)
ss46630>dTrpA1(M)
SS46630>dTrpA1 (F)
empty>dTrpA1 (M)
empty>dTrpA1(F)
Sleep (mins/30mins)
ZT0
ZT12
CT0
CT12
CT0
CT12
Day 1 (LD21)
B. ZT 0-12
C. ZT 12-24
Males
Females
Sleep (mins)
SS46630/dTRPA1
empty/dTrpA1
SS46630/+
Day 2 (DD 29)
D. CT 0-12
E. CT 12-24
Day 3 (DD21)
F. CT 0-12
G. CT 12-24

Figure S4: Activation of OA-VPM3 neurons in DD suppresses sleep in males and female flies

A. Sleep plots of male and female flies (SS46630>dTRPA1 and empty>dTRPA1) for 3 days. Environmental conditions are described, and flies were tested after 3 days of 12h L and 12hr D entrainment. Day 1(LD, 21), Day 2 (DD, 29) and Day 3 (DD, 21).

B, C: Sleep duration of male and female flies on Day 1 (12 hr bins) for indicated genotypes with empty-split or SS46630 (VPM3 driver) expressing UAS-dTRPA1 and SS46630/+.

D, E: Sleep duration of male and female flies on Day 2 (12 hr. bins) for indicated genotypes with empty-split or SS46630 (VPM3 driver) expressing UAS-dTRPA1 and SS46630/+.

F, G : Sleep duration of male and female flies on Day 3 (12 hr. bins) for indicated genotypes with empty-split or SS46630 (VPM3 driver) expressing UAS-dTRPA1 and SS46630/+.

Mean ± SEM is shown and comparisons are made using Kruskal-Wallis test followed by Dunns multiple comparisons test. Statistical significances are indicated as ∗$p < 0.05$; ∗∗$p < 0.01$; and ∗∗∗$p < 0.001$. Control (SS46630/+ and empty/dTRPA1) groups were compared with SS46630/dTRPA1. Number of flies ranged 41-62 for male and female flies.

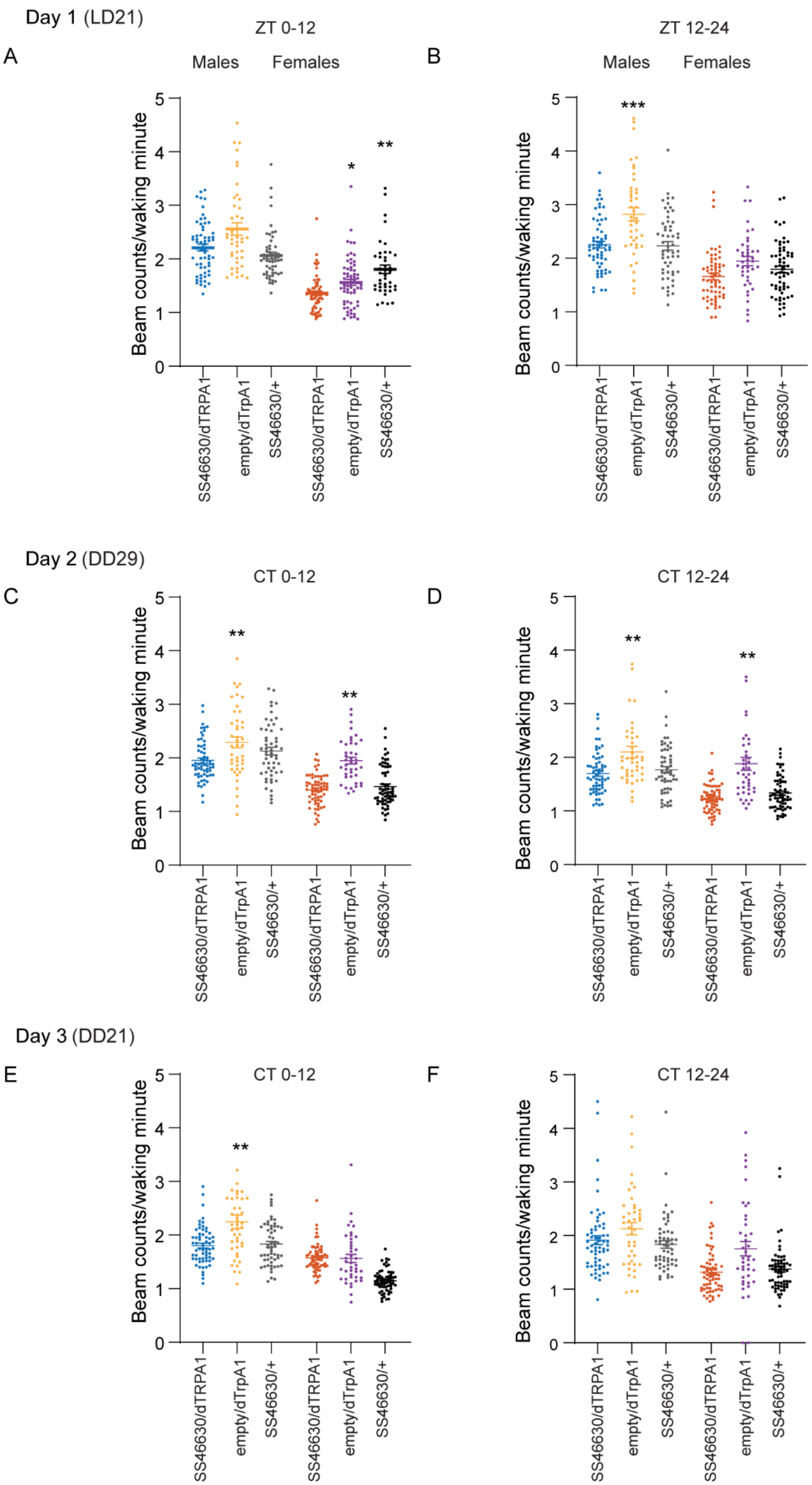
Day 1 (LD21)
A
ZT 0-12
Males
Females
B
ZT 12-24
Males
Females
Day 2 (DD29)
C
CT 0-12
D
CT 12-24
Day 3 (DD21)
E
CT 0-12
F
CT 12-24
Beam counts/waking minute
0
1
2
3
4
5
SS46630/dTRPA1
empty/dTrpA1
SS46630/+
SS46630/dTRPA1
empty/dTrpA1
SS46630/+
*
**
***

Figure S5: Activity (beam counts/waking minute) in flies with OA-VPM3 activation during DD

A-F: Activity of flies (SS46630/+, SS46630/dTRPA1 and empty/dTRPA1) measured on day 1,2 and 3. Environmental conditions of each day are indicated. Mean ± SEM is shown and comparisons are made using Kruskal-Wallis test followed by Dunns multiple comparisons test. Statistical significances are indicated as $*p < 0.05$; $**p < 0.01$; and $***p < 0.001$. Control (SS46630/+ and empty/dTRPA1) groups were compared with SS46630/dTRPA1.

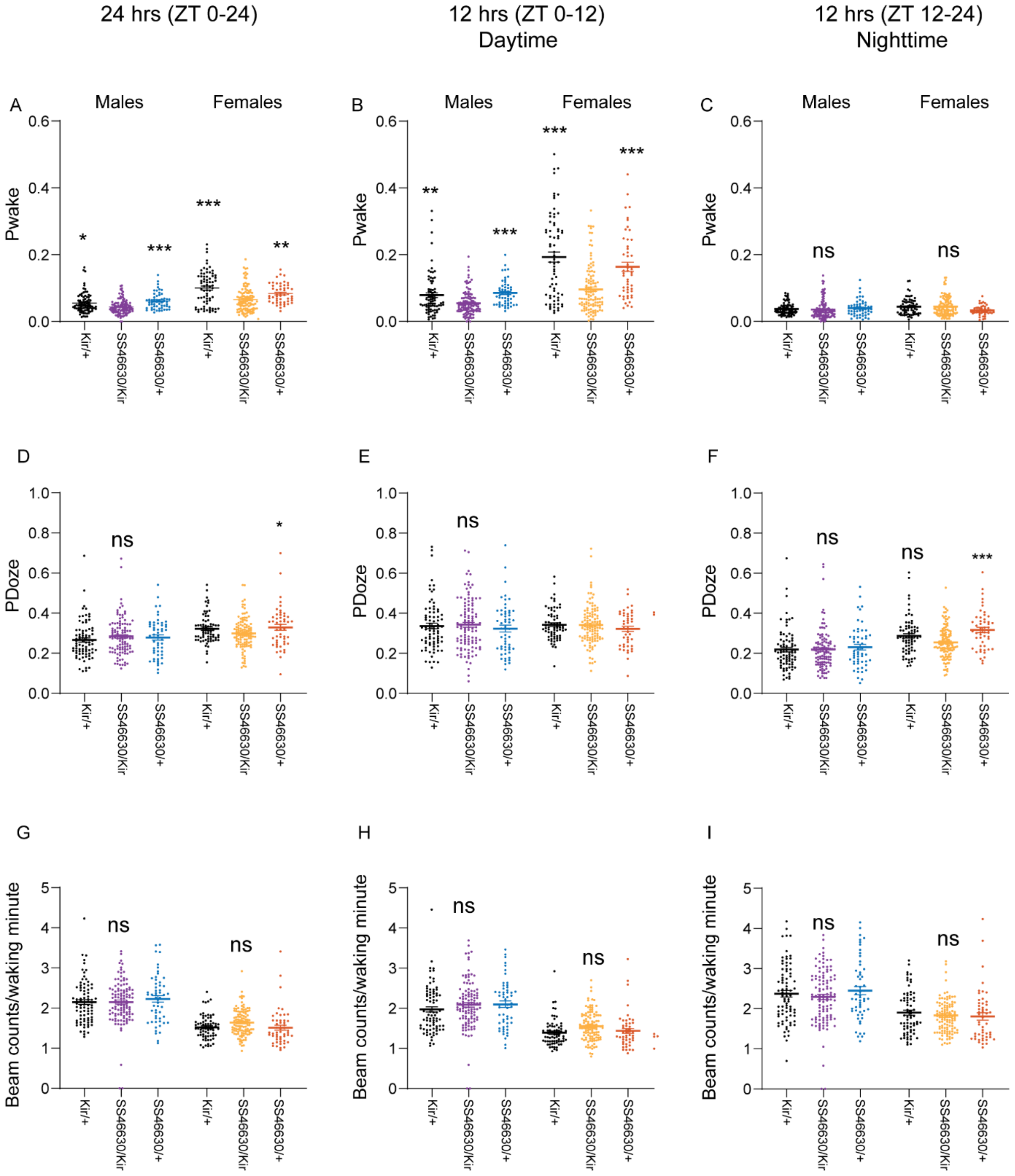

Figure S6. P(wake), P(doze), and activity in flies with OA-VPM3 neuronal inhibition

A-C. Mean P(wake) for 24 hr, daytime and nighttime duration in male and female flies of the genotype SS46630>UAS-Kir2.1, Kir2.1/+ and SS46630/+.

D-F. Mean P(doze) for 24 hr, daytime and nighttime duration in male and female flies of the genotype SS46630>UAS-Kir2.1, Kir2.1/+ and SS46630/+.

G-I. Mean Activity (beam counts/waking minute) for 24 hr, daytime and nighttime duration in male and female flies of the genotype SS46630>UAS-Kir2.1, Kir2.1/+ and SS46630/+.

For A-I, Mean ± SEM is shown, and comparisons are made using Kruskal-Wallis test followed by Dunns multiple comparisons test. Statistical significances are indicated as $*p < 0.05$; $**p < 0.01$; $***p < 0.001$; ns, not significant. Control (SS46630/+ and Kir2.1/+) groups were compared with SS46630/dTRPA1.

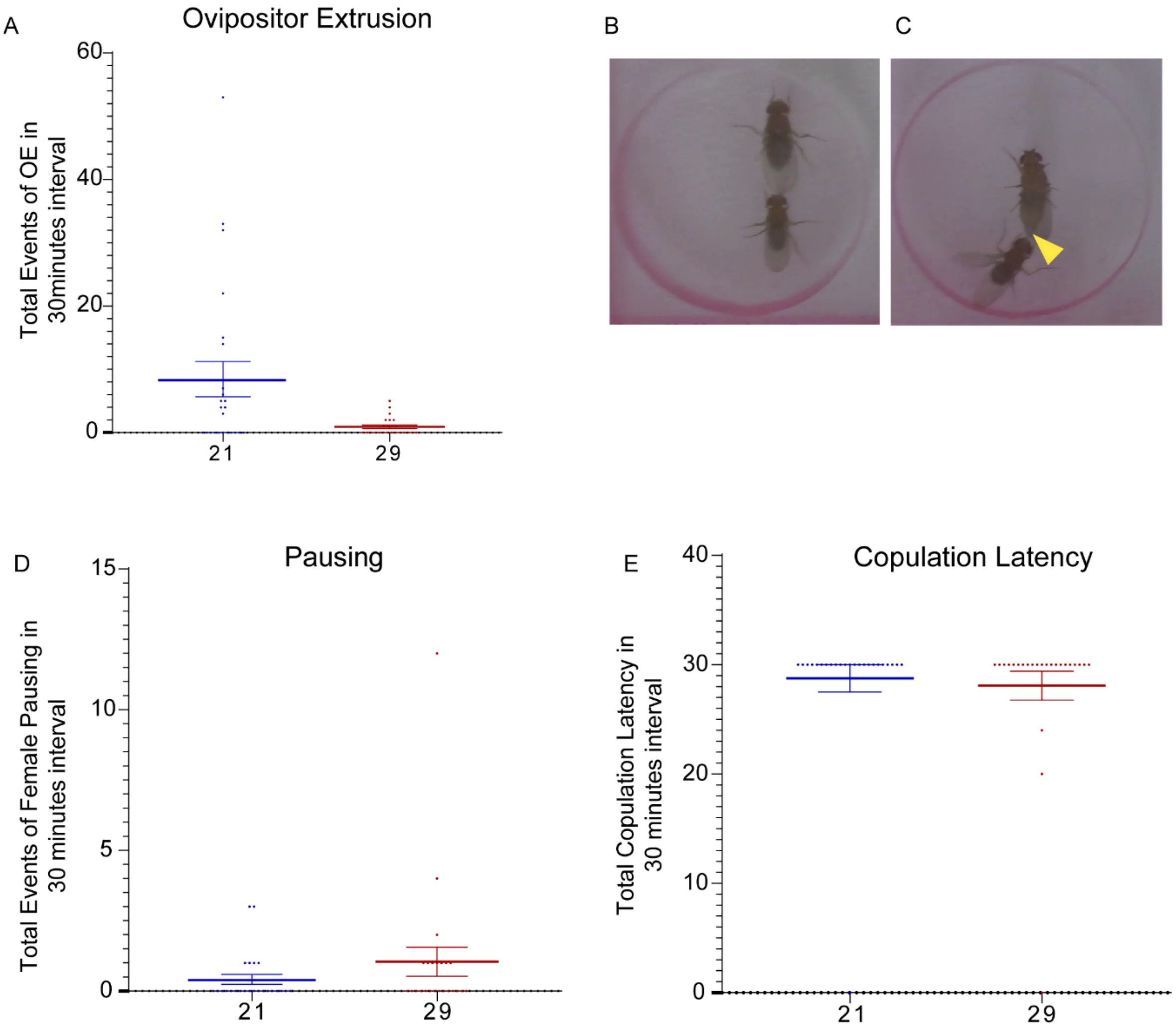

Figure S7: Female receptivity is not altered by OA-VPM3 activation.

A. Number of ovipositor extrusion events during 30-minute recordings at 21 and 29ºC degrees. n=24 flies. SS46630>dTRPA1 female flies were tested with tester males.

B and C. Sample image from recording showing pausing and ovipositor extrusion events

D. Number of pausing events during 30-minute recording at 21 and 29ºC degrees. n=24 flies.

E. Number of pausing events during 30-minute recordings at 21 and 29ºC degrees. n=24 flies.

For each genotype two conditions were compared using unpaired t-test (Mann-Whitney U test) and receptivity experiments were conducted between ZT 2-8.

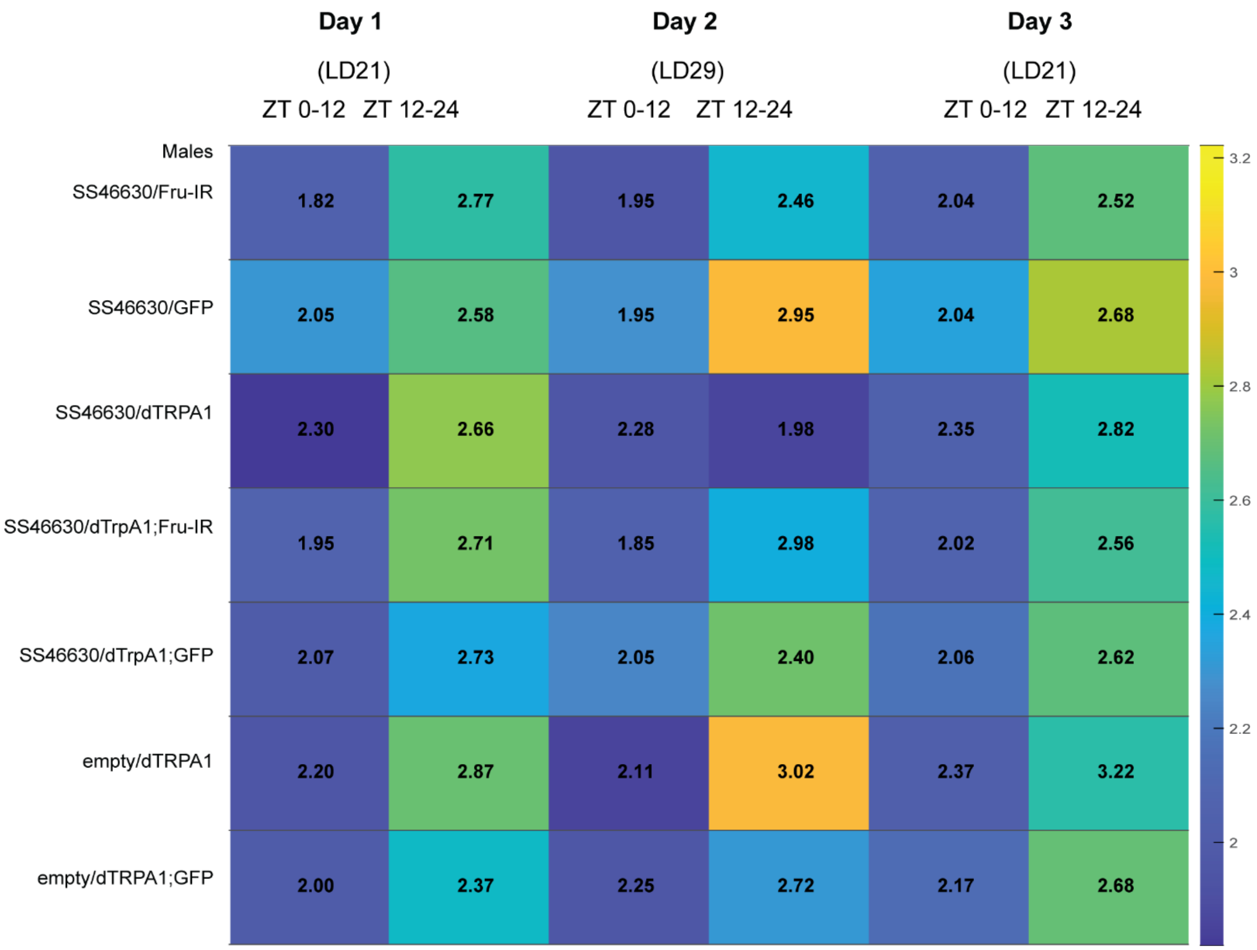

Figure S8: Activity (beam counts/waking minute) of flies expressing Fru-IR, GFP, dTRPA1 and combinations

Heat map showing activity of SS46630/Fru-IR, SS46630/GFP, SS46630/dTRPA1, SS46630/dTRPA1; Fru-IR, SS46630/dTRPA1; GFP, empty/dTRPA1 and empty/dTRPA1;GFP male flies. Data block represents 12 hours on days 1, 2 and 3.

A.
B.
C.

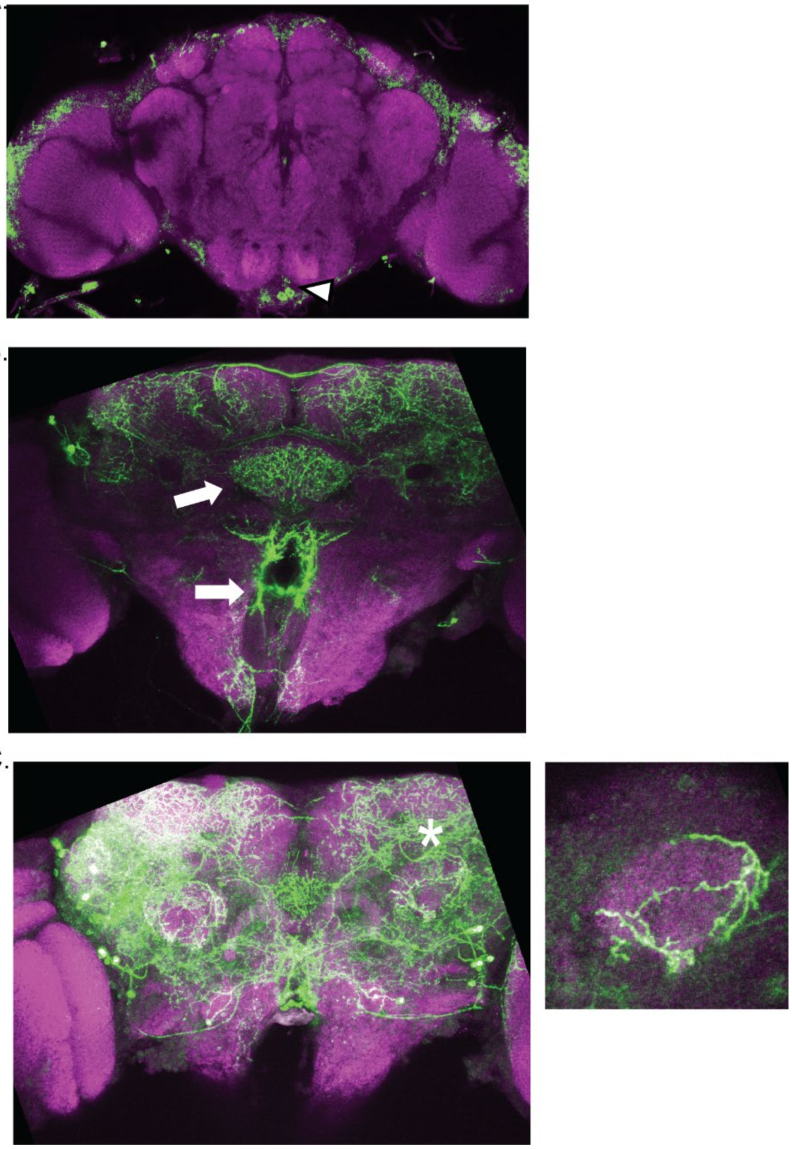

Figure S9: VPM3-LexA expression in male brains.

A. Whole-mount brain immunostained with anti-GFP and nc82. Arrowhead indicates cell bodies of OA-VPM3 neurons.

B. Arrows show fb innervations and projections from sez to dorsal regions of the brain. The image is a z-stack of anterior projections (~ 40um thickness).

C. Image z-stack of posterior projections (Left panel). Left panel shows projections in the calyx.

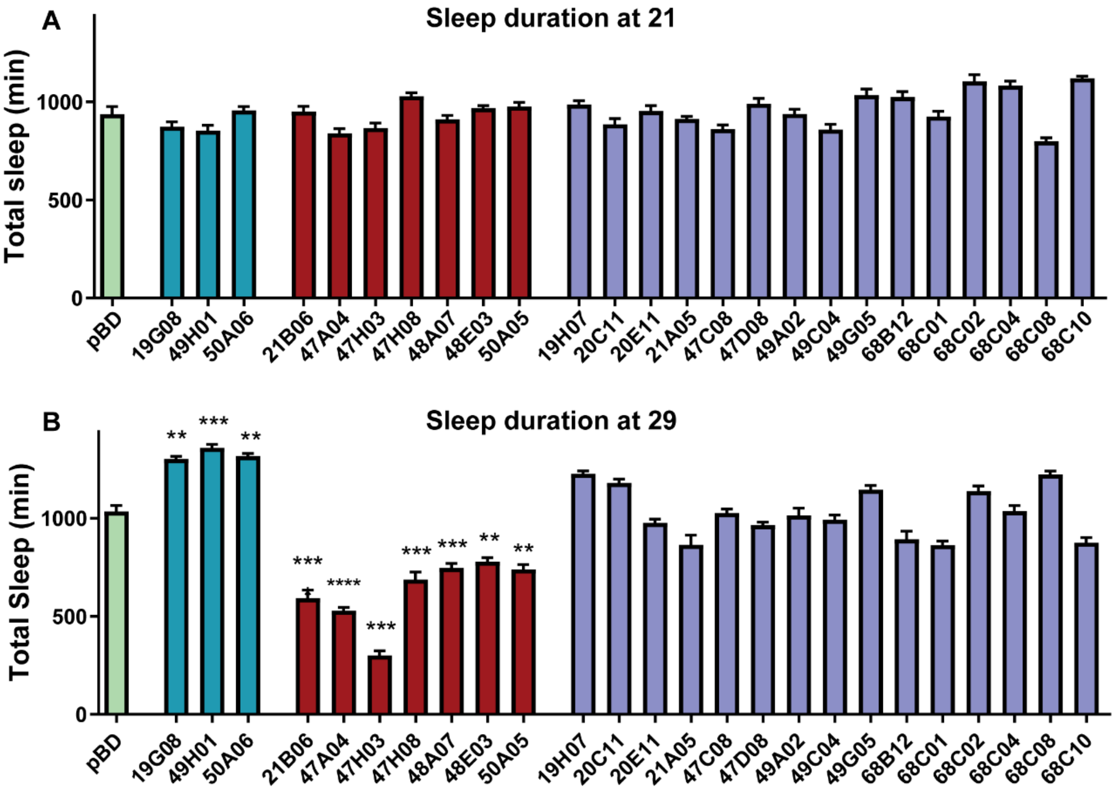

OAMB-GAL4s: 47A04, 47C08, 47D08, 47H03, 47H08, 48A07, 48E03, 49A02, 49C04, 49G05, 49H01, 50A05, 50A06
Octβ1R-GAL4s: 19G08, 19H07, 20E11, 21C11, 21A05, 21B06
Octβ2R-GAL4s: 68C04, 68C08, 68C10
Octβ3R-GAL4s: 68C01, 68C02, 68B12

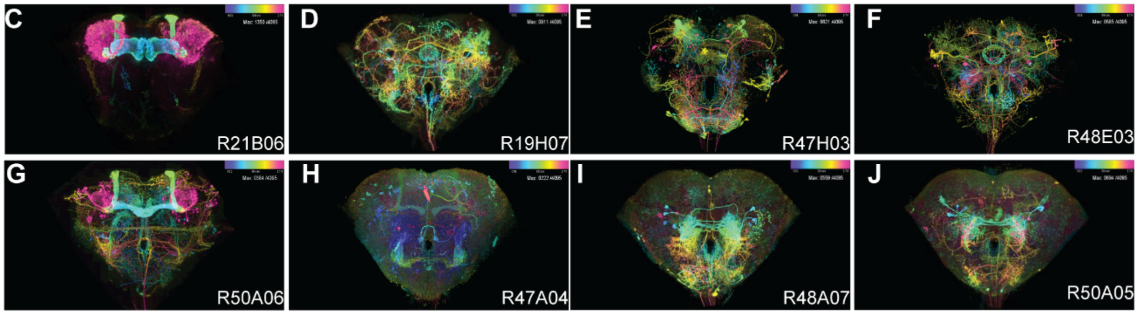

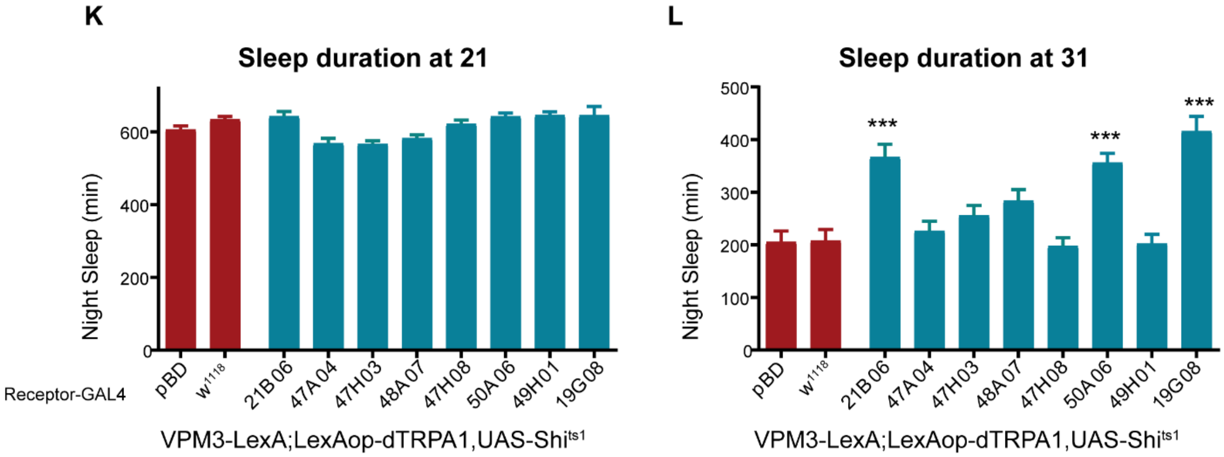

Figure S10: Behavioral and anatomical screening of OA Receptor-GAL4 lines for sleep phenotypes

A and B. Flies expressing UAS-dTRPA1 and 25 octopamine receptor GAL4 lines were tested at 21 and 29ºC to identify receptor-GAL4 lines involved in sleep regulation.). Empty-GAL4 expressing dTRPA1 was used as a genotypic control and data is represented as mean and SEM and comparisons are made by Kruskal-Wallis test followed by Dunns multiple comparisons test. Statistical significances are indicated as $*p < 0.05$; $**p < 0.01$; $***p < 0.001$; ns, not significant. For each genotype no data was excluded, and data collected represents 2 or more independent trials.

C-J. Whole-mount brain immunostaining of 8 octopamine receptor GAL4 lines with altered sleep on activation and sparse expression patterns. OA receptor GAL4 lines are expressing. Maximal intensity projection of the central brain was made from original z stack files obtained from https://flweb.janelia.org/cgi-bin.

K. Nighttime sleep duration (day 1, baseline at 21) of flies expressing VPM3-LexA; LexAop-dTRPA1 and X-Gal4; UAS-Shi^{ts1}. X-Gal4 represents 8 OA receptor GAL4 lines (R21B06, R47A04, R47H03, R48A07, R47H08, R50A06, R49H01 and R19G08). Controls include empty-Gal4 (pBD) and w1118.

L. Nighttime sleep duration (day 2, 29 degrees)) of flies expressing VPM3-LexA; LexAop-dTRPA1 and X-Gal4; UAS-Shi^{ts1}. X-Gal4 represents 8 OA receptor GAL4 lines (R21B06, R47A04, R47H03, R48A07, R47H08, R50A06, R49H01 and R19G08). Controls include empty-Gal4 and w1118.

For K and L, and data is represented as mean and SEM and comparisons are made by Kruskal-Wallis test followed by Dunns multiple comparisons test. Statistical significances are indicated as $*p < 0.05$; $**p < 0.01$; $***p < 0.001$; ns, not significant. For each genotype no data was

excluded, and data collected represents 2 or more independent trials number of tested flies ranged from 28-40 flies.

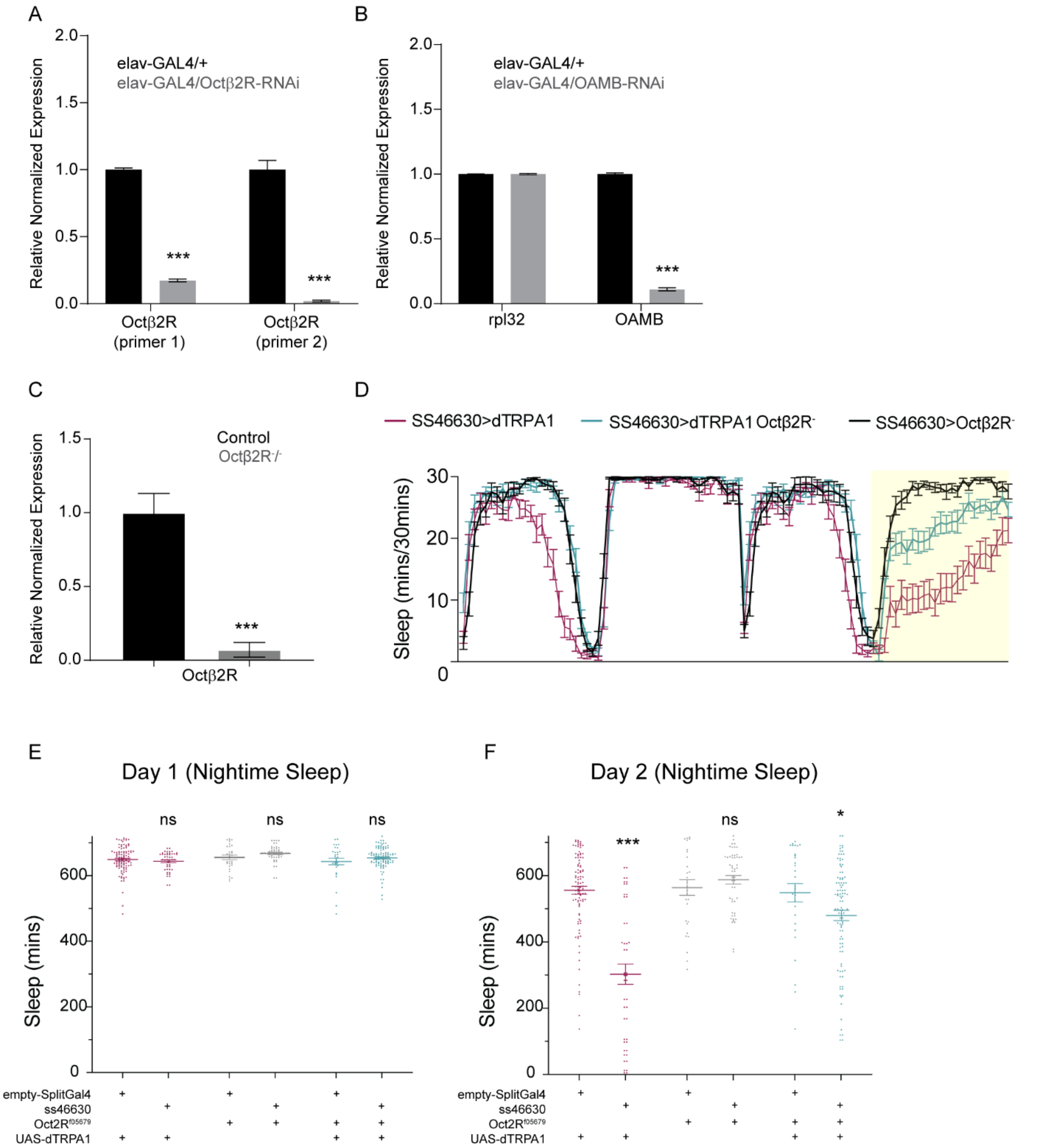

Figure S11: Octβ2R receptor is required for sleep suppression induced by OA-VPM3 activation.

A and B. Quantitative RT–PCR of Octβ2R and OAMB in flies with RNAi mediated pan-neuronal knockdown. Samples were compared using Mann Whitney U test and $***p < 0.001$.

C. Quantitative RT–PCR of Octβ2R mutant and controls. Samples were compared using Mann Whitney U test and $***p < 0.001$.

D. Representative sleep profile (mean and sem) of OA-VPM3>dTRPA1 flies in Octβ2R null and wild type background. The data represents baseline day 1 and day at 21°C (daytime and nighttime) and day 2 (daytime at 21 ^{o}C and nighttime at 29 ^{o}C).

E and F. Sleep profile of flies (SS46630>dTRPA1, SS46630> Octβ2R- and SS46630>dTRPA1, Octβ2R-). Day 1 and Day 2 represent daytime and nighttime sleep at 21 and 29°C respectively and Day 1 represents daytime and nighttime sleep at 21 and Day 2 represents daytime at 21 and nighttime at 29 degrees respectively. Groups were compared made by Kruskal-Wallis test followed by Dunns multiple comparisons test. Statistical significances are indicated as $*p < 0.05$; $**p < 0.01$; $***p < 0.001$; ns, not significant.
